# CD163^+^ macrophages monitor enhanced permeability at the blood–dorsal root ganglion barrier

**DOI:** 10.1084/jem.20230675

**Published:** 2023-12-20

**Authors:** Harald Lund, Matthew A. Hunt, Zerina Kurtović, Katalin Sandor, Paul B. Kägy, Noah Fereydouni, Anais Julien, Christian Göritz, Elisa Vazquez-Liebanas, Maarja Andaloussi Mäe, Alexandra Jurczak, Jinming Han, Keying Zhu, Robert A. Harris, Jon Lampa, Jonas Heilskov Graversen, Anders Etzerodt, Lisbet Haglund, Tony L. Yaksh, Camilla I. Svensson

**Affiliations:** 1 Department of Physiology and Pharmacology, Center for Molecular Medicine, Karolinska Institutet, Stockholm, Sweden; 2 Kancera AB, Karolinska Institutet Science Park, Stockholm, Sweden; 3 Department of Medicine, Rheumatology Unit, Center for Molecular Medicine, Karolinska Institutet, Karolinska University Hospital, Stockholm, Sweden; 4 Department of Cell and Molecular Biology, Karolinska Institutet, Stockholm, Sweden; 5 Department of Immunology, Genetics, and Pathology, Rudbeck Laboratory, Uppsala University, Uppsala, Sweden; 6 Department of Clinical Neuroscience, Center for Molecular Medicine, Karolinska Institutet, Karolinska University Hospital, Stockholm, Sweden; 7 Department of Molecular Medicine, University of Southern Denmark, Odense, Denmark; 8 Department of Biomedicine, Aarhus University, Aarhus, Denmark; 9 Division of Orthopaedic Surgery, Department of Surgery, McGill University, Montreal, Canada; 10 Department of Anesthesiology, University of California, San Diego, CA, USA

## Abstract

In dorsal root ganglia (DRG), macrophages reside close to sensory neurons and have largely been explored in the context of pain, nerve injury, and repair. However, we discovered that most DRG macrophages interact with and monitor the vasculature by sampling macromolecules from the blood. Characterization of the DRG vasculature revealed a specialized endothelial bed that transformed in molecular, structural, and permeability properties along the arteriovenous axis and was covered by macrophage-interacting pericytes and fibroblasts. Macrophage phagocytosis spatially aligned with peak endothelial permeability, a process regulated by enhanced caveolar transcytosis in endothelial cells. Profiling the DRG immune landscape revealed two subsets of perivascular macrophages with distinct transcriptome, turnover, and function. CD163^+^ macrophages self-maintained locally, specifically participated in vasculature monitoring, displayed distinct responses during peripheral inflammation, and were conserved in mouse and man. Our work provides a molecular explanation for the permeability of the blood–DRG barrier and identifies an unappreciated role of macrophages as integral components of the DRG-neurovascular unit.

## Teaser

The blood–DRG barrier is composed of endothelial cells with high permeability and is monitored by a subset of CD163^+^ perivascular macrophages, a process that is arteriovenously zonated. Vascular monitoring is abrogated by interfering with caveolar transcytosis in endothelial cells or by depleting CD163^+^ macrophages.

## Graphical Abstract

**Figure:**
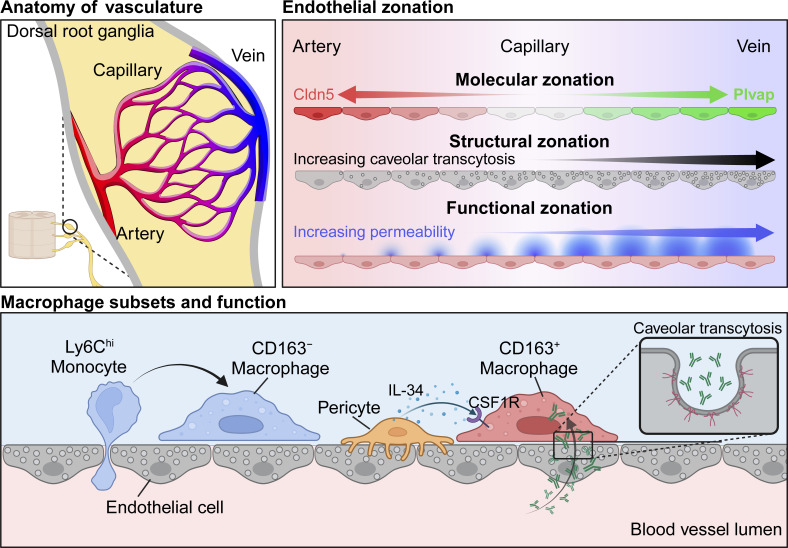

## Introduction

Tissue-resident macrophages are multifunctional and highly plastic immune cells that are found in every organ of the body. A core function of macrophages shared across most organs is to act as tissue sentinels and scavengers by phagocytosing cellular debris and orchestrating tissue repair. In addition, macrophages participate in more complex, tissue-specific processes, as diverse as the wiring of neural networks, production of blood vessels and bone, as well as turnover of dying cells or essential proteins in the liver, spleen, and lung (Mass et al., 2023; Park et al., 2022). It is now understood that such tissue-specialized functions are the result of distinct molecular programs induced by microenvironmental cues (cell-contacts and secreted molecules) in their tissues of residence (Gosselin et al., 2014; Lavin et al., 2014; Bonnardel et al., 2019). Another factor contributing to macrophage variability in different organs relates to their ontogeny. During development, organs are colonized by embryonic macrophages derived from precursors in the yolk sac or fetal liver that can self-sustain throughout life (Ginhoux et al., 2010; Gomez Perdiguero et al., 2015; Hoeffel et al., 2015; Schulz et al., 2012). By contrast, there are subpopulations of macrophages identified in most tissues that require constant replenishment from bone marrow–derived monocytes (Dick et al., 2022; Shaw et al., 2018; Molawi et al., 2014).

Macrophages in the central nervous system (CNS) are well studied, where parenchymal microglia and border-associated macrophages, residing in perivascular, meningeal, and choroid plexus niches, are recognized (Mildenberger et al., 2022). In the peripheral sensory nervous system, however, aspects such as transcriptional heterogeneity, ontogeny, microenvironmental regulation, and homeostatic function are only starting to be explored (Kolter et al., 2020; Ydens et al., 2020; Wang et al., 2020a). Macrophages associate with the entire length of sensory neurons, from the distal peripheral nerve endings to the proximal processes entering the spinal column. Macrophages are also prominent in dorsal root ganglia (DRG; Zigmond and Echevarria, 2019), which are segmentally organized collections of sensory neuron cell bodies located alongside the spinal cord. Transcriptional differences between macrophages residing in these different locations are evident, indicating that they are a product of their endoneurial microenvironment (Wang et al., 2020a). DRG macrophages increase in number during a range of neuropathic conditions (Peng et al., 2016; Zhang et al., 2016; van der Vlist et al., 2022), which may promote the development of pain (Yu et al., 2020; Raoof et al., 2021) or help resolve it (van der Vlist et al., 2022; Singh et al., 2022). However, the homeostatic functions of macrophages in the DRG have remained largely overlooked. Moreover, heterogeneity within the macrophage pool, which is recognized in virtually every organ investigated, has not been systematically examined in the DRG.

The axons of sensory neurons are protected from circulating toxic molecules and pathogens by the blood–nerve barrier (BNB), which shares functional and morphological features with the blood–brain barrier (BBB), including low levels of transcytosis, high expression of tight junction proteins, and lack of endothelial fenestrae (Ubogu, 2020). The neuronal cell bodies in the DRG, however, are not only supplied by a denser vascular bed than the axons in the peripheral nerve (Jimenez-Andrade et al., 2008); the endothelial cells within DRGs also have significantly higher permeability (Jacobs et al., 1976; Kobayashi and Yoshizawa, 2002; Olsson, 1968; Arvidson, 1979). Despite the long-standing appreciation for these properties, an understanding of the mechanisms regulating endothelial permeability at the blood–DRG barrier remains lacking. Furthermore, the role of macrophages in this context has remained unexplored. Breakdown of endothelial barrier integrity occurs and contributes to disease progression in a range of conditions affecting the BBB (Profaci et al., 2020) and the BNB (Ubogu, 2020; Richner et al., 2019). The elevated permeability observed in DRG endothelium makes the DRG susceptible to neurotoxic molecules, autoantibodies, and infectious agents, and is likely a major cause of sensory ganglionopathies, conditions leading to sensory loss or painful manifestations (Amato and Ropper, 2020). Characterizing the cellular components of the vascular niche and understanding how endothelial barrier integrity is maintained in the DRG therefore remain important areas of research.

In this study, we describe a dense network of perivascular macrophages residing in sensory ganglia, tasked with monitoring the permeable blood–DRG barrier. Mechanistically, we found that this process was driven by elevated caveolar transcytosis in endothelial cells coupled to a self-sustained and highly phagocytic macrophage subset. Our work identifies a novel immunovascular unit that has implications for understanding BNB homeostasis, disease, and therapy.

## Results

### DRG macrophages monitor the vasculature

**Figure 1.**
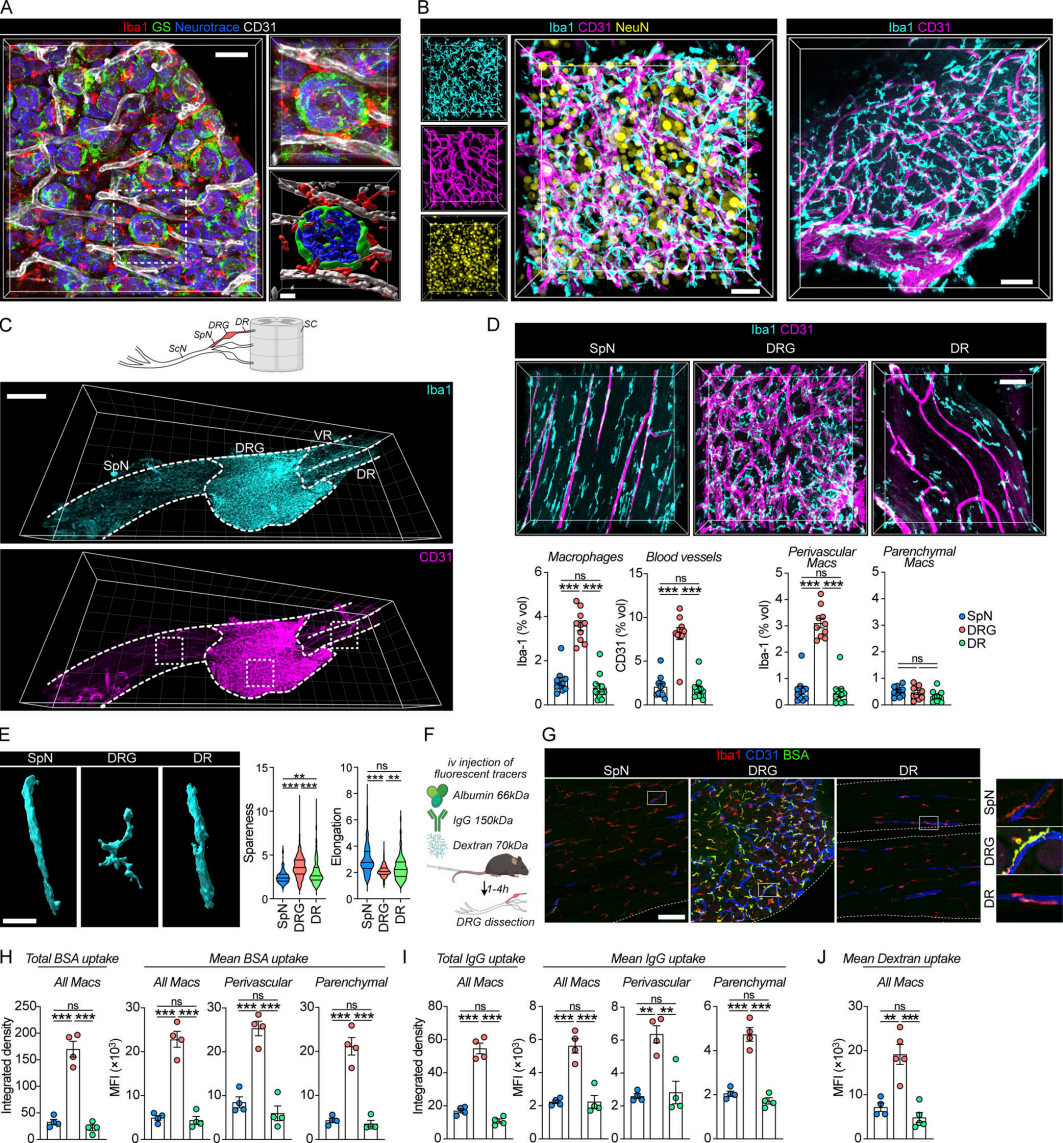
**DRG macrophages interact with the vasculature. (A)** Representative immunostaining of neurons, SGCs, endothelial cells, and macrophages in naive DRGs. Scale bar, 40 and 10 μm (inset). **(B)** 3D confocal images of DRG whole mounts to visualize neurons, macrophages, and endothelial cells. Scale bar, 30 μm (left) and 80 μm (right). **(C)** 3D confocal images of iDISCO-cleared whole mounts of DRGs with attached SpN and DRs and ventral roots (VR) stained with Iba1 and CD31. Scale bar, 400 μm. **(D)** Confocal Z-stacks of boxed areas in C and quantifications of macrophage and vascular density performed in indicated regions. *n* = 10 (SpN), 10 (DRG), 11 (DR) mice. Tukey’s multiple comparisons test. ***P < 0.001; ns, not significant. Scale bar, 40 μm. **(E)** Morphology of perivascular macrophages as measured by their spareness (high value indicates spider-like shape) and elongation (high value indicates cigar-like shape). Data are mean values of individual macrophages from *n* = 10 (SpN), 10 (DRG), 11 (DR) mice. Tukey’s multiple comparisons test. **P < 0.01, ***P < 0.001; ns, not significant. Scale, bar 10 μm. **(F)** Experiment schematic of i.v tracer injections. Created with https://BioRender.com. **(G and H)** (G) Representative confocal images (scale bar, 100 μm) and (H) quantification of BSA-A647 (20 mg/kg) uptake in Iba1^+^ macrophages 1 h after i.v injection. *n* = 4 mice/group. The experiment was performed twice. Tukey’s multiple comparisons test. ***P < 0.001; ns, not significant. **(I)** Uptake of goat IgG-A488 (4 mg/kg) in Iba1^+^ macrophages 4 h after i.v injection. *n* = 4 mice/group. The experiment was performed twice. Tukey’s multiple comparisons test. **P < 0.01, ***P < 0.001; ns, not significant. **(J)** Uptake of 70 kD dextran-TMR (10 mg/kg) in Iba1^+^ macrophages 2 h 45 min after i.v injection. The experiment was performed twice. Tukey’s multiple comparisons test. **P < 0.01, ***P < 0.001; ns, not significant.

Macrophage functions are shaped by and tailored to their cellular microenvironment, known as the “macrophage niche” (Guilliams et al., 2020). To characterize the macrophage niche in the DRG at a cellular level, we costained macrophages (Iba1^+^) and major cell types in the DRG, including neurons (Neurotrace^+^), and satellite glial cells (SGC, GS^+^), which wrap around the neuronal soma (Hanani and Spray, 2020). Given the high degree of vascularization in the DRG (Jimenez-Andrade et al., 2008), we also stained endothelial cells. This analysis revealed that macrophages were positioned in the space between SGCs and endothelial cells (Fig. 1 A), often making close contact with endothelial cells (Fig. 1 B), prompting us to further explore the interaction between macrophages and endothelial cells in the DRG.

**Figure S1.**
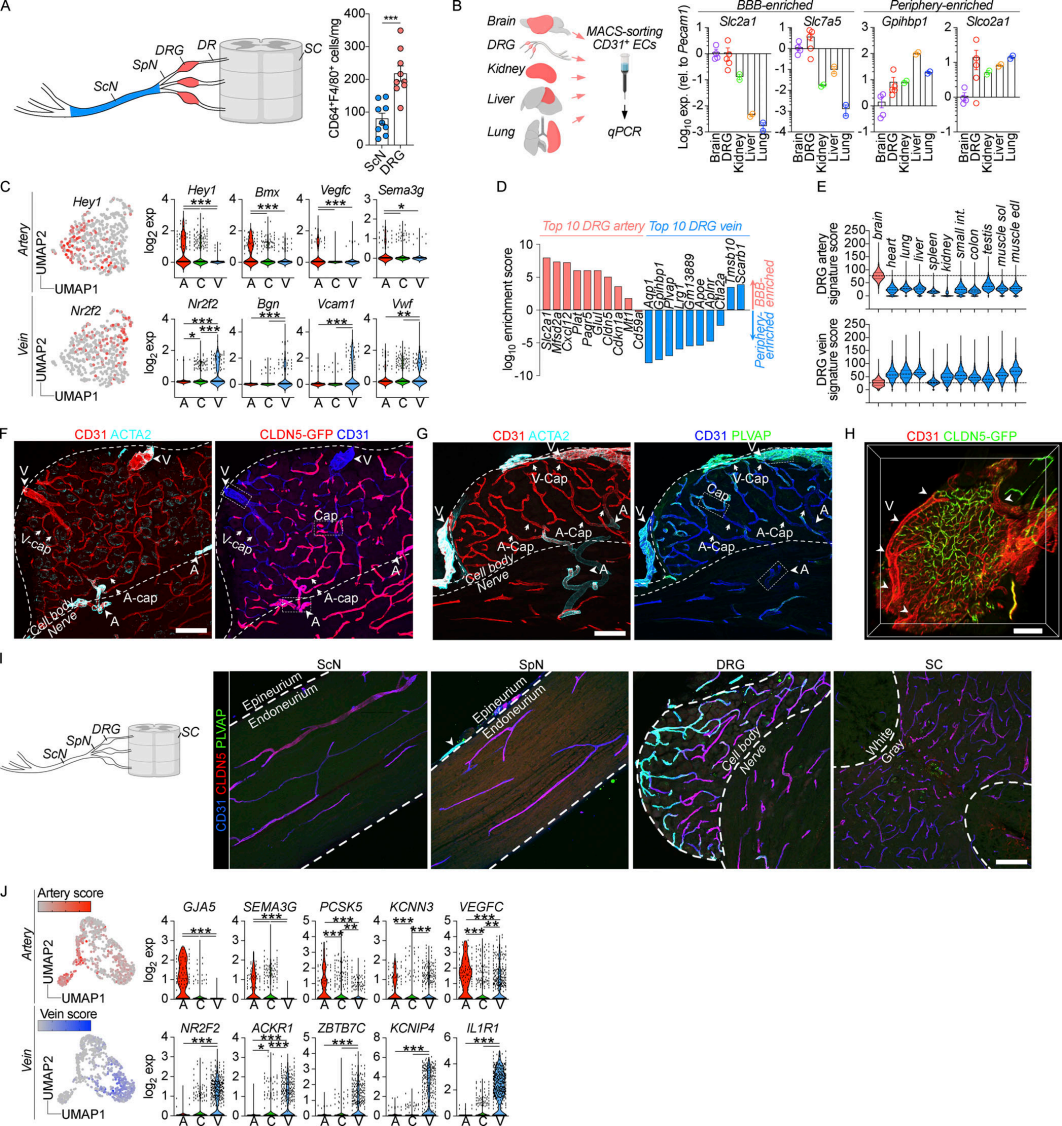
**Additional arteriovenous zonation characteristics of DRG endothelium. (A)** Flow cytometry–based quantification of CD64^+^F4/80^+^ DRG or ScN macrophages, normalized to wet weight of tissue. *n* = 3 experiments pooled, three mice/exp. Student’s unpaired *t* test. ***P < 0.001. **(B)** qPCR of MACS-enriched CD31^+^ endothelial cells (ECs) from indicated organs, normalized to brain. *n* = 4 (brain), 5 (DRG), 2 (kidney, liver, lung). Illustration created with https://BioRender.com. **(C)** Expression of artery and vein signature genes across mouse DRG endothelial clusters (Avraham et al., 2020): artery (A), capillary (C), vein (V). Tukey’s multiple comparisons test. *P < 0.05, **P < 0.01, ***P < 0.001. **(D)** Enrichment of DRG vein or artery signature genes in BBB or peripheral endothelial cells (Munji et al., 2019). DRG artery genes are enriched in BBB, whereas DRG vein genes are enriched in peripheral endothelium. **(E)** Expression of DRG vein or artery signature genes in endothelial cells isolated from indicated organs (Kalucka et al., 2020). DRG artery genes are enriched in brain endothelial cells, and DRG vein genes are enriched in several peripheral endothelial types. **(F)** CLDN5-GFP expression in entire DRG section from *Cldn5*^GFP/+^ mouse, visualizing cropped areas in Fig. 2 H. Scale bar, 100 μm. **(G)** Immunostaining of PLVAP in entire DRG section, visualizing cropped areas in Fig. 2 I. Scale bar, 100 μm. **(H)** Whole-mount imaging of native GFP expression in DRG from *Cldn5*^GFP/+^ mice, confirming absence of CLDN5 expression in large veins (arrowheads) and venous capillaries. Scale bar, 200 μm. **(I)** CLDN5 and PLVAP immunostaining in indicated tissues. Outside of the DRG, PLVAP^+^CLDN5^−^ vessels are only present in epineurial blood vessels. ScN and SpN endoneurial vessels are PLVAP^−^CLDN5^+^. Scale bar, 100 μm. **(J)** Artery and vein signature gene expression in human DRG endothelial clusters (Avraham et al., 2022). Tukey’s multiple comparisons test. *P < 0.05, **P < 0.01, ***P < 0.001.

We next stained Iba1^+^ macrophages and CD31^+^ endothelial cells in DRG wholemounts followed by iDISCO tissue clearing and 3D-confocal imaging (Fig. 1 C). DRGs were dissected with the spinal nerve (SpN) and dorsal root (DR) attached, which allowed comparisons of macrophages across these three tissue regions. Quantification of macrophage volume showed a significant increase in the DRG compared with adjacently located SpN and DR (Fig. 1 D). This finding was confirmed using flow cytometry of enzymatically digested tissues, showing that the DRG contained a higher number of CD64^+^F4/80^+^ macrophages than did the sciatic nerve (ScN) per weight of tissue (Fig. S1 A). Comparison of the level of vascularization similarly demonstrated a fourfold increase in the DRG compared with the SpN and DR (Fig. 1 D), which supports previous findings (Jimenez-Andrade et al., 2008). We then investigated the spatial relationship between endothelial cells and macrophages, observing that macrophages that were in direct contact with the abluminal side of endothelial cells (perivascular) were approximately sixfold more prevalent in the DRG than in the DR or SpN (Fig. 1 D and Video 1). Macrophages that did not make contact with the vasculature (parenchymal) were similar in volume across the three tissue regions (Fig. 1 D). The morphology of perivascular DRG macrophages was also distinct, coiling around vessels and displaying a more tortuous shape than their SpN and DR counterparts. SpN and DR perivascular macrophages had a more elongated shape, extending along blood vessels parallel to the axons (Fig. 1 E). Our data thus far demonstrate a local increase in perivascular macrophages specifically in the DRG.

**Video 1.** **Visualization of macrophage-endothelial contact in iDISCO cleared DRG whole mount stained for neurons (NeuN), endothelial cells (CD31), and macrophages (Iba1), followed by segmentation of perivascular macrophages. **30 frames per second.

**Figure S2.**
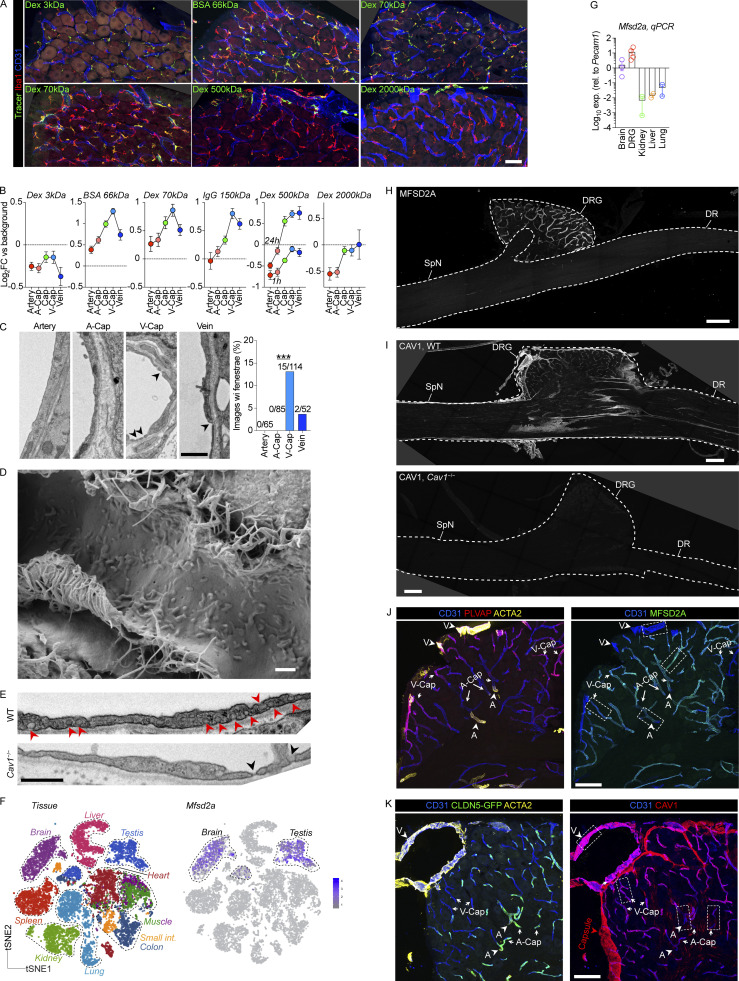
**Additional analysis of tracer uptake, ultrastructure, and caveolar transcytosis in DRG endothelium. (A)** Low-magnification images of Iba1 and CD31 stained DRG sections from mice injected with indicated i.v tracers. All tracers readily accumulate inside macrophages. Images related to analyses in Fig. 3 A and panel B. Scale bar, 50 μm. **(B)** Uptake of indicated i.v injected tracers in DRG endothelial cells, separated by vessel segments. The following tracers, doses, and circulation times were used: 3 kD dextran-TMR (25 mg/kg, 1 h, *n* = 4 mice), BSA-A647 (5 mg/kg, 1 h, *n* = 4 mice), 70 kD dextran-TMR (25 mg/kg, 1 h, *n* = 9 mice), goat anti rabbit IgG-A488 (4 mg/kg, 4 h, *n* = 4 mice), 500 kD dextran-FITC (25 mg/kg, 1 h or 24 h, *n* = 4 mice), 2,000 kD dextran-FITC (25 mg/kg, 24 h, *n* = 4 mice). Values are mean of individual macrophages, normalized to tissue background. **(C)** Analysis of endothelial fenestrae (arrowheads) in TEM images from indicated DRG vessel segments. Total images per vessel segment are indicated above each bar. *n* = 4 mice. Fisher’s exact test. ***P < 0.001. Scale bar, 1 μm. **(D)** Scanning electron micrograph of luminal surface of DRG v-cap with an apparent scarcity of endothelial fenestrae. Scale bar, 500 nm. **(E)** Representative TEM images of v-caps from WT and *Cav1*^−/−^ mice, showing the absence of caveolar vesicles (red arrowhead), but presence of fenestrae (black arrowhead) in *Cav1*^−/−^ mice. Scale bar, 500 nm. **(F)** *Mfsd2a* expression across endothelial cells isolated from indicated organs showing expression restricted to brain and testis (Kalucka et al., 2020). **(G)** *Mfsd2a* expression in MACS-enriched CD31^+^ endothelial cells from indicated organs, normalized to brain. *n* = 3 (brain), 4 (DRG), 2 (kidney, liver, lung). **(H)** MFSD2A immunostaining in DRG section, demonstrating that MFSD2A is absent in SpN and DR. Scale bar, 200 μm. **(I)** CAV1 immunostaining in entire DRG section from WT and *Cav1*^−/−^ mice, demonstrating antibody specificity. Scale bar, 200 μm. **(J)** Representative MFSD2A immunostaining in DRG section, visualizing cropped areas in Fig. 3 D. Scale bar, 100 μm. **(K)** Representative CAV1 immunostaining in DRG section, visualizing cropped areas in Fig. 3 D. Scale bar, 100 μm. A, artery; V, vein.

Given the well-described capacity of low and high molecular weight compounds to permeate the blood–DRG barrier (Jacobs et al., 1976; Kobayashi and Yoshizawa, 2002; Olsson, 1968; Arvidson, 1979), we next addressed whether macrophages had the capacity to phagocytose circulating molecules. To that end, we intravenously (i.v) injected mice (Fig. 1 F) with either fluorescently labeled albumin (BSA, 66 kD) or IgG (150 kD), the two most abundant proteins in plasma. Sacrificing animals within 1 h (BSA) or 4 h (IgG) revealed that both proteins readily accumulated inside DRG macrophages (Fig. S2 A), and quantification across sensory nerve regions demonstrated a five- to eightfold (BSA, Fig. 1, G and H) or two- to fivefold (IgG, Fig. 1 I) higher uptake in DRG macrophages compared with SpN or DR macrophages. To exclude that uptake was not due to a higher proportion of perivascular macrophages in the DRG, we analyzed perivascular and parenchymal macrophages separately, which resulted in similar results (Fig. 1, H and I). To substantiate these findings, we next injected 70 kD dextran, a branched glucan used clinically as a plasma substitute that displays minimal extravasation across the healthy BBB (Armulik et al., 2010). Similarly to our IgG and BSA injections, dextran uptake was elevated in DRG macrophages compared with SpN and DR macrophages (Fig. 1 J). These results demonstrate that the majority of DRG macrophages interact with the vasculature and actively phagocytose endogenous and exogenous molecules from circulation.

### The DRG vasculature displays both barrier and permeable properties and has a conserved arteriovenous distribution

While differences in vascular permeability between the blood–nerve and blood–ganglion barrier are recognized (Reinhold and Rittner, 2020), an in-depth molecular understanding of this phenomenon is lacking. We hypothesized that increased vascular permeability could at least partly explain the level of circulating protein uptake in DRG macrophages, and we next sought to better characterize DRG endothelial cells. Guided by a previous report (Munji et al., 2019), we first analyzed whether DRG endothelial cells expressed markers specific to the BBB or peripheral endothelium. This revealed that DRG endothelial cells expressed high levels of the glucose transporter *Slc2a1* as well as the amino acid transporter *Slc7a5 *(Fig. S1 B), both of which are specific to the BBB (Munji et al., 2019; Kalucka et al., 2020). DRG endothelial cells also expressed *Gpihbp1*, involved in lipid metabolism, and the prostaglandin transporter *Slco2a1*, which are normally expressed in kidney, liver, and lung endothelium but are absent in the BBB (Fig. S1 B). These results indicated that DRG blood vessels expressed both peripheral and CNS-specific markers and further suggested transcriptomic heterogeneity across the DRG endothelial population.

**Figure 2.**
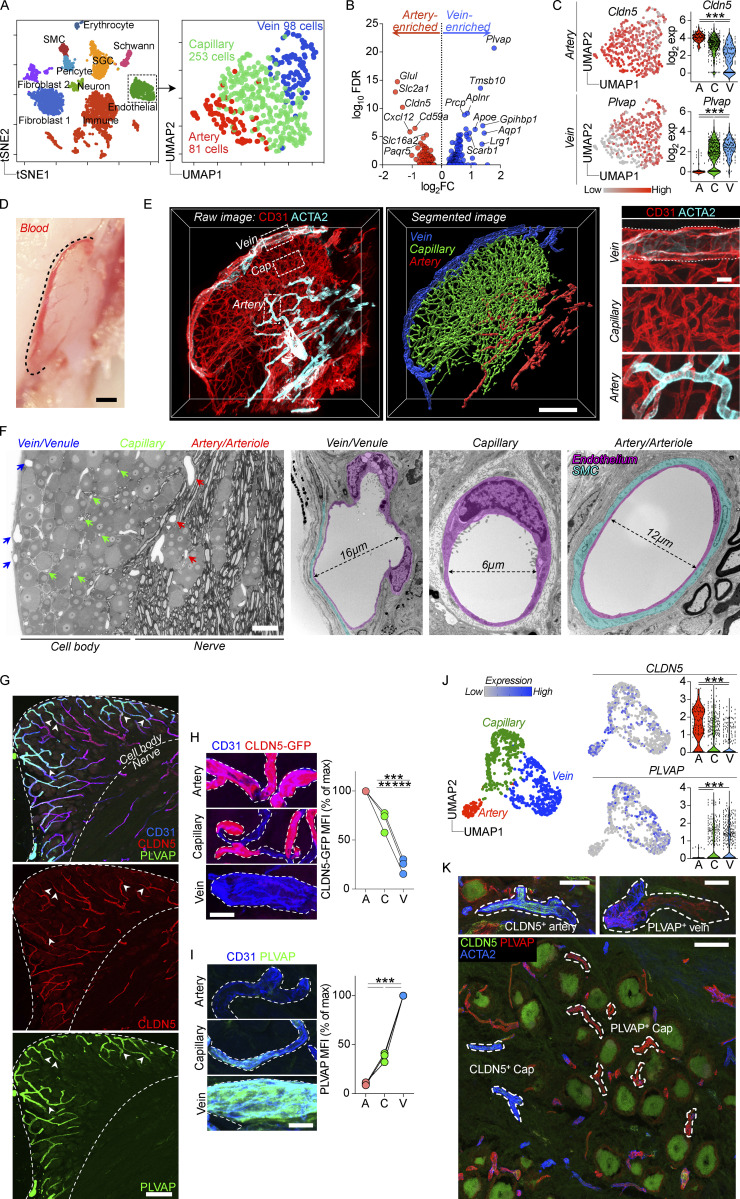
**DRG vasculature has dual identity. (A)** Reclustering of published scRNA-seq data of 432 DRG endothelial cells from mouse (Avraham et al., 2020), displaying clusters with artery, capillary, and vein identity. **(B)** Differential gene expression between artery and vein clusters using Venice algorithm. **(C)** *Cldn5* (artery; A) and *Plvap* (vein; V) expression across clusters. Tukey’s multiple comparisons test. ***P < 0.001. **(D)** Photomicrograph of undissected L5 DRG from unperfused mouse illustrating blood-filled vasculature. Scale bar, 200 μm. **(E)** Whole-mount imaging and iDISCO tissue clearing of CD31 and ACTA2 stained lumbar DRG. 3D reconstruction and vessel segment identification using Imaris. Scale bar, 200 and 20 μm (inset). **(F)** Identification of vessel segments in ultrathin DRG sections by TEM, based on their anatomical localization. Scale bar, 50 μm. **(G)** Immunolocalization of CLDN5 and PLVAP expression to CD31^+^ DRG endothelial cells, displaying minimal overlap. Arrows indicate PLVAP/CLDN5 breakpoints. Scale bar, 100 μm. **(H and I)** (H) CLDN5-GFP (*Cldn5*^GFP/+^ mice) and (I) PLVAP expression in DRG vessel segments, normalized to % of max. *n* = 3 mice. Tukey’s multiple comparisons test. **P < 0.01, ***P < 0.001. Scale bar, 20 μm. C, capillary. **(J)** Reclustering snRNA-seq data of 777 endothelial nuclei from human DRGs from five donors (Avraham et al., 2022) and the expression of *CLDN5* and *PLVAP* across artery, capillary, and vein clusters. Tukey’s multiple comparisons test. ***P < 0.001. **(K)** Immunostaining of CLDN5 and PLVAP in human DRG sections. Scale bars, 100 μm.

Progressive transcriptomic changes along the arteriovenous axis, a process defined as “zonation,” have been recognized in several endothelial beds using single-cell RNA sequencing (scRNA-seq; Vanlandewijck et al., 2018; Kalucka et al., 2020). To address transcriptomic zonation in the DRG vasculature, we reclustered a publicly available mouse scRNA-seq dataset (Avraham et al., 2020), which included 432 DRG endothelial cells. Three clusters of endothelial cells were identified (Fig. 2 A), which we annotated as artery, capillary, and vein, respectively, based on expression of well-established (Vanlandewijck et al., 2018; Kalucka et al., 2020; Trimm and Red-Horse, 2022) artery- (*Hey1*, *Bmx*, *Vegfc*, *Sema3g*) and vein-specific markers (*Nr2f2*, *Bgn*, *Vcam1*, *Vwf*) in the two clusters that occupied the extremes of the Uniform Manifold Approximation and Projection (UMAP; Fig. S1 C). Differential gene expression further revealed that the artery cluster was characterized by high expression of *Cldn5*, *Slc2a1*, and *Mfsd2a *(Fig. 2 B), which are all highly enriched in brain endothelial cells (Fig. S1, D and E). *Cldn5* encodes a tight junction protein that maintains BBB integrity (Greene et al., 2019) and *Mfsd2a* is a transporter of essential fatty acids required for proper brain development and function (Nguyen et al., 2014; Ben-Zvi et al., 2014). The vein cluster displayed high expression of *Plvap*, *Aqp1*, *Gpihbp1*, and *Lrg1*, all being enriched in peripheral endothelial beds (Fig. S1, D and E). *Plvap* encodes a protein restricted to endothelial fenestrae, transendothelial channels, and caveolar vesicles; structures involved in microvascular permeability (Guo et al., 2016). All these markers, including *Plvap* and *Cldn5*, displayed a zonated expression profile, peaking in either arteries or veins and gradually decreasing or increasing along the arteriovenous axis (Fig. 2 C).

We next wanted to validate *Cldn5* and *Plvap* at the protein level. To anatomically identify the three vessel segments, we stained CD31 and smooth muscle actin (ACTA2) in DRG wholemounts and used 3D-confocal imaging to reconstruct the DRG vasculature. This analysis revealed a conserved anatomical distribution, where arteries entered the neuron-rich region from the nerve fiber, giving rise to a capillary bed that was subsequently collected into veins on the DRG surface (Fig. 2, D and E; and Video 2). Transmission electron microscopy (TEM) further validated the anatomical location of these vessel segments (Fig. 2 F). We explored the expression of the two top artery and vein markers, *Cldn5* and *Plvap*, at the protein level. Costaining in DRG sections revealed that both markers were restricted to endothelial cells, but with minimal overlap, PLVAP being expressed in superficially located vessels and CLDN5 in those closer to the nerve fiber (Fig. 2 G). Using *Cldn5*^GFP/+^ mice, we could confirm high expression of CLDN5 in ACTA2^+^ arteries, intermediate expression in capillaries, and complete absence in large veins on the DRG surface (Fig. 2 H and Fig. S1, F and H). This pattern was reversed for PLVAP, which displayed the highest expression in large veins and intermediate expression in capillaries, whereas ACTA2^+^ arteries were completely devoid of PLVAP expression (Fig. 2 I and Fig. S1 G). The presence of CLDN5^−^PLVAP^+^ capillaries entering the endoneurium appeared unique to the DRG as it was not observed in the SpN or ScN, where CLDN5^−^PLVAP^+^ vessels were restricted to the epineurium (Fig. S1 I). Our results reveal that the DRG arteriovenous tree has a predictable anatomical localization and is distinguished by a gradual phenotypic shift characterized by loss of barrier properties and gain of permeability properties.

**Video 2.** **Visualization of vessel segments (artery, capillary, and vein) in iDISCO cleared DRG whole mounts by staining for endothelial cells (CD31) and SMCs (ACTA2). **30 frames per second.

### Arteriovenous zonation is conserved in human DRG vasculature

Human DRGs have higher in vivo blood perfusion rates than the SpN as measured by functional magnetic resonance imaging (Godel et al., 2016), suggesting that DRG endothelial permeability is a feature shared between mouse and man. However, the vasculature has not previously been studied in detail in human DRGs. We thus sought to address whether human DRGs displayed a similar profile as in mouse. We first analyzed a recently published single nucleus RNA sequencing (snRNA-seq) dataset of human DRGs (Avraham et al., 2022), which included one cluster of 777 *PECAM1*^+^ (encoding CD31) endothelial cells. Subclustering of endothelial cells revealed three distinct clusters that could be assigned vein, capillary, and artery annotations, based on expression of artery- (*GJA5*, *SEMA3G*, and *VEGFC*) and vein- (*NR2F2*, *KCNIP4*, and *IL1R1*) enriched markers identified in other organs (Fig. S1 J; Chen et al., 2022; Yang et al., 2022; Trimm and Red-Horse, 2022). Exploring the top barrier and permeability markers identified in mouse DRG endothelial cells, *CLDN5* and *PLVAP*, revealed that both genes were zonated and similarly enriched in arteries and veins, respectively (Fig. 2 J). Using DRG tissues collected from human organ donors (Table S1), we validated CLDN5 expression in ACTA2^+^ arteries and arterioles and its absence from veins and most capillaries supplying the neuronal soma-rich region (Fig. 2 K). Consistent with the snRNA-seq data, PLVAP was absent in arteries, but stained most capillaries supplying the neuronal soma-rich areas, as well as large veins in the capsule (Fig. 2 K). This indicates that the human DRG vasculature is also characterized by a dual identity with barrier-type arteries and highly permeable veins.

### Macrophage monitoring of DRG vasculature is arteriovenously zonated and requires caveolar transcytosis

**Figure 3.**
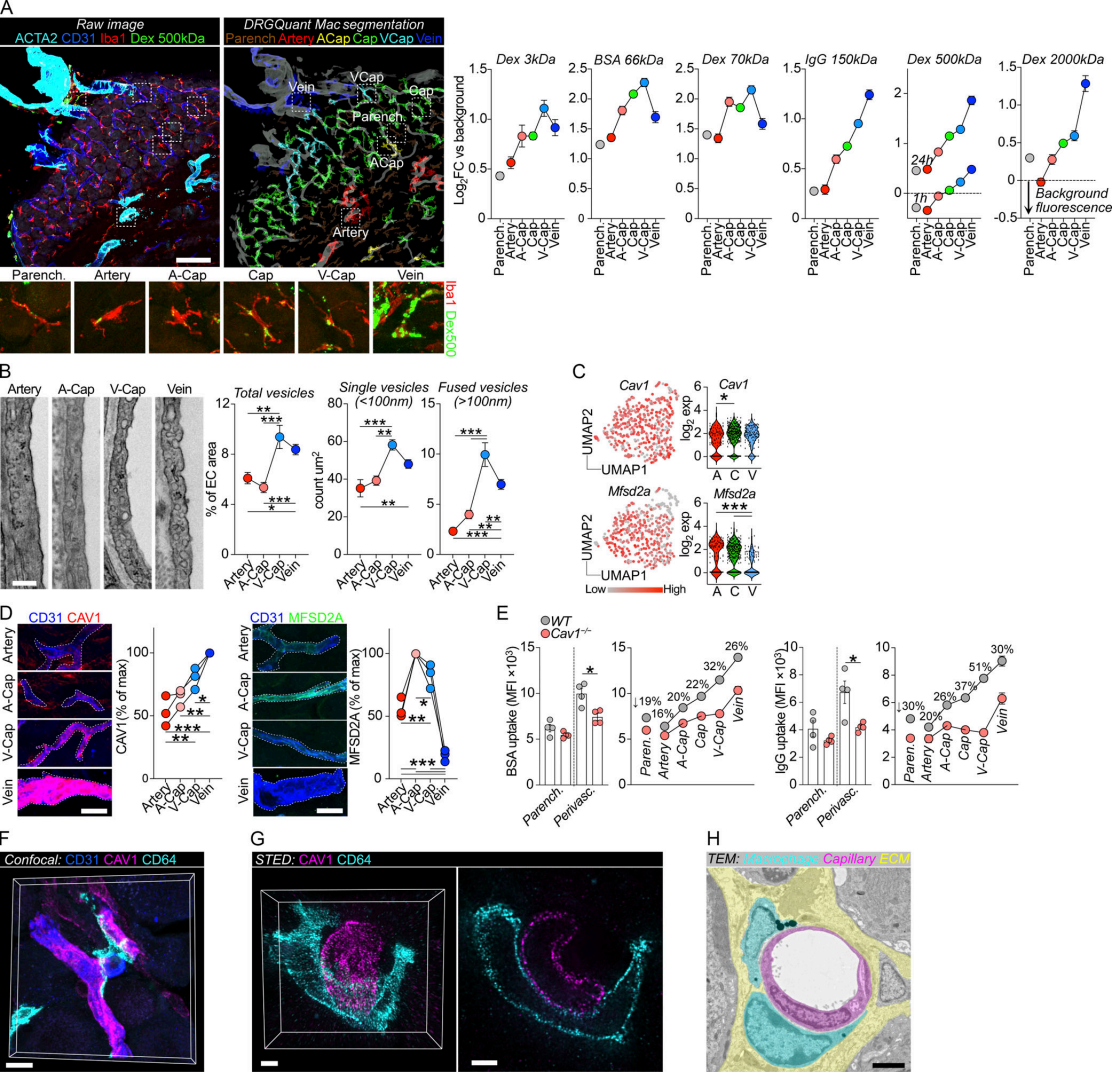
**Macrophage monitoring is arteriovenously zonated and requires caveolar vesicles. (A)** Machine-learning-based spatial mapping of Iba1^+^ macrophages to DRG vessel segments and analysis of the uptake of indicated i.v-injected tracers using the DRGQuant algorithm. The following tracers, doses, and circulation times were used: 3 kD dextran-TMR (25 mg/kg, 1 h, *n* = 4 mice), BSA-A647 (5 mg/kg, 1 h, *n* = 4 mice), 70 kD dextran-TMR (25 mg/kg, 1 h, *n* = 9 mice), goat anti rabbit IgG-A488 (4 mg/kg, 4 h, *n* = 4 mice), 500 kD dextran-FITC (25 mg/kg, 1 h or 24 h, *n* = 4 mice), 2,000 kD dextran-FITC (25 mg/kg, 24 h, *n* = 4 mice). Values are the mean of individual macrophages, normalized to tissue background. Scale bar, 100 μm. **(B)** Machine-learning-based quantifications of endothelial vesicles (<100 nm diameter) in high-resolution TEM images of indicated DRG vessel segments. Data are mean of 50 (artery), 51 (a-cap), 42 (v-cap), and 120 (vein) images from *n* = 2 mice. Tukey’s multiple comparisons test. *P < 0.05, **P < 0.01, ***P < 0.001. Scale bar, 250 nm. **(C)** Expression of *Cav1* and *Mfsd2a* mRNA in DRG vessel segments. Tukey’s multiple comparisons test. *P < 0.05, ***P < 0.001. **(D)** CAV1 and MFSD2A immunostaining in DRG sections and expression across vessel segments normalized to % of max. *n* = 3 mice. Tukey’s multiple comparisons test. *P < 0.05, **P < 0.01, ***P < 0.001. Scale bars, 25 μm. A, artery; V, vein. **(E)** Macrophage uptake of i.v injected BSA-A488 (1 mg/ml) and Goat IgG-A647 (1 mg/ml, 2 h circulation) in WT and *Cav1*^−/−^ mice (*n* = 4/group). Bar graphs are quantification of uptake in parenchymal and perivascular Iba1^+^ macrophages. Line-connected graphs are quantifications of perivascular macrophages across endothelial vessel segments. Percentages indicate the reduction in macrophage uptake between WT to *Cav1*^−/−^ mice at each vessel segment. Multiple unpaired *t* test with Holm Sidak correction. *P < 0.05. The experiment was performed twice. **(F)** Confocal image of CD64^+^ macrophage and CAV1^+^ DRG capillary. Scale bar, 10 μm. **(G)** STED-captured Z-stack (left) and one Z-layer (right) of CD64^+^ macrophage in contact with CAV1^+^ capillary. Scale bar, 2 μm. **(H)** TEM image of macrophage-endothelial contact. Scale bar, 2 μm.

The zonated proteogenomic profile of the DRG vasculature next led us to address whether endothelial permeability was variable along the arteriovenous axis. Using DRGQuant, a machine-learning-based algorithm that we recently developed to analyze DRG macrophages in tissue sections (Hunt et al., 2022), we performed spatial mapping of macrophages along the arteriovenous tree (Fig. 3 A). We i.v injected mice with a series of macromolecules of different sizes including dextrans, albumin, and IgG, ranging from 3 to 2,000 kD. All tracers readily and preferentially accumulated inside macrophages (Fig. S2 A). We therefore analyzed macrophage-mediated uptake and organized the data based on which vessel segment the macrophages contacted. This demonstrated a gradual increase in macrophage uptake from arteries to veins, consistently peaking in either venous capillaries (v-cap) or veins (Fig. 3 A). While the mean uptake in endothelial cells was lower than in macrophages, the uptake across vessel segments mirrored that in macrophages (Fig. S2 B).

The DRG vasculature thus displayed arteriovenously zonated permeability, which correlated with the presence of CLDN5^−^PLVAP^+^ endothelial cells. As PLVAP is restricted to endothelial fenestrae, transendothelial channels, and caveolae (Guo et al., 2016), we next sought to quantify the presence of these substructures across DRG vessel segments using TEM. The presence of fenestral openings in the DRG vasculature has been reported (Jacobs et al., 1976; Arvidson, 1979; Anzil et al., 1976; Kobayashi and Yoshizawa, 2002), and we did identify fenestrae which were virtually restricted to v-caps (Fig. S2 C), although their numbers were limited (only 0.06% of endothelial lining). The overall scarcity of fenestrae was further confirmed by scanning electron microscopy (SEM) visualization of the inner lumen of DRG vessels (Fig. S2 D). We did not observe any transendothelial channels in the DRG vasculature (data not shown). Using higher-magnification TEM images, we instead observed that small endothelial vesicles (∼100 nm), likely caveolar vesicles, were ubiquitous in the DRG vasculature. Machine-learning-based image quantification revealed a significantly higher presence of such vesicles in v-caps and veins compared with arteries and arterial capillaries (a-caps; Fig. 3 B). We confirmed that these vesicles were caveolar vesicles as they were absent in the DRG endothelium from *Cav1*^−/−^ mice (Fig. S2 E), which cannot form caveolae (Parton et al., 2020). However, fenestrae were still present in DRG v-caps from *Cav1*^−/−^ mice (Fig. S2 E). To further understand the regulation of caveolae across DRG vessel segments, we quantified expression of CAV1, the main scaffolding protein required for caveolae assembly. We also explored expression of MFSD2A, an inhibitor of caveolar transcytosis (Andreone et al., 2017), which is restricted to barrier endothelium in the CNS, testis (Fig. S2 F), and retina (Wang et al., 2020b). We recorded high levels of Mfsd2a mRNA and protein in DRG endothelial cells (Fig. S2, G and H) and that mRNA expression gradually decreased from arteries to veins (Fig. 3 C). Protein levels of MFSD2A were similarly zonated, reaching a peak in a-caps and then gradually decreasing to negligible levels in veins (Fig. 3 D and Fig. S2 J). *Cav1* was not zonated at the mRNA level (Fig. 3 C), but its protein level displayed an opposite pattern to that of MFSD2A, increasing gradually from arteries to veins (Fig. 3 D and Fig. S2, I and K). This suggested that CAV1 expression may be regulated by MFSD2A in the DRG vasculature, which is observed at the BBB and the blood–retinal barrier (Wang et al., 2020b; Andreone et al., 2017).

To investigate if caveolar transcytosis was required for macrophage monitoring of DRG endothelium, we used *Cav1*^−/−^ mice and injected BSA and IgG into the tail vein. DRG macrophage uptake of i.v injected IgG and BSA were both significantly reduced in *Cav1*^−/−^ mice compared with WT controls (Fig. 3 E). When perivascular macrophages were spatially mapped along the arteriovenous axis and analyzed based on which vessel segment they contacted, the largest difference between WT and *Cav1*^−/−^ mice was noted in v-caps (Fig. 3 E), which is consistent with the high level of caveolar vesicles observed in this vessel segment. This indicated that perivascular macrophages were ingesting material passing across endothelial cells via caveolar vesicles. In support of this hypothesis, using confocal and stimulated emission depletion (STED) microscopy, we observed that DRG macrophages made direct contact with the cell membrane of CAV1^+^ v-caps (Fig. 3, F and G). TEM further confirmed this notion, showing that macrophages and endothelial cells made direct cell-to-cell contact in this location (Fig. 3 H). Taken together, our data demonstrate a structural zonation across the DRG vasculature that spatially aligns with its permeability. Furthermore, caveolar transcytosis is at least partly required for monitoring of the vasculature by DRG macrophages.

### Two molecularly distinct subsets of macrophages inhabit the DRG

**Figure 4.**
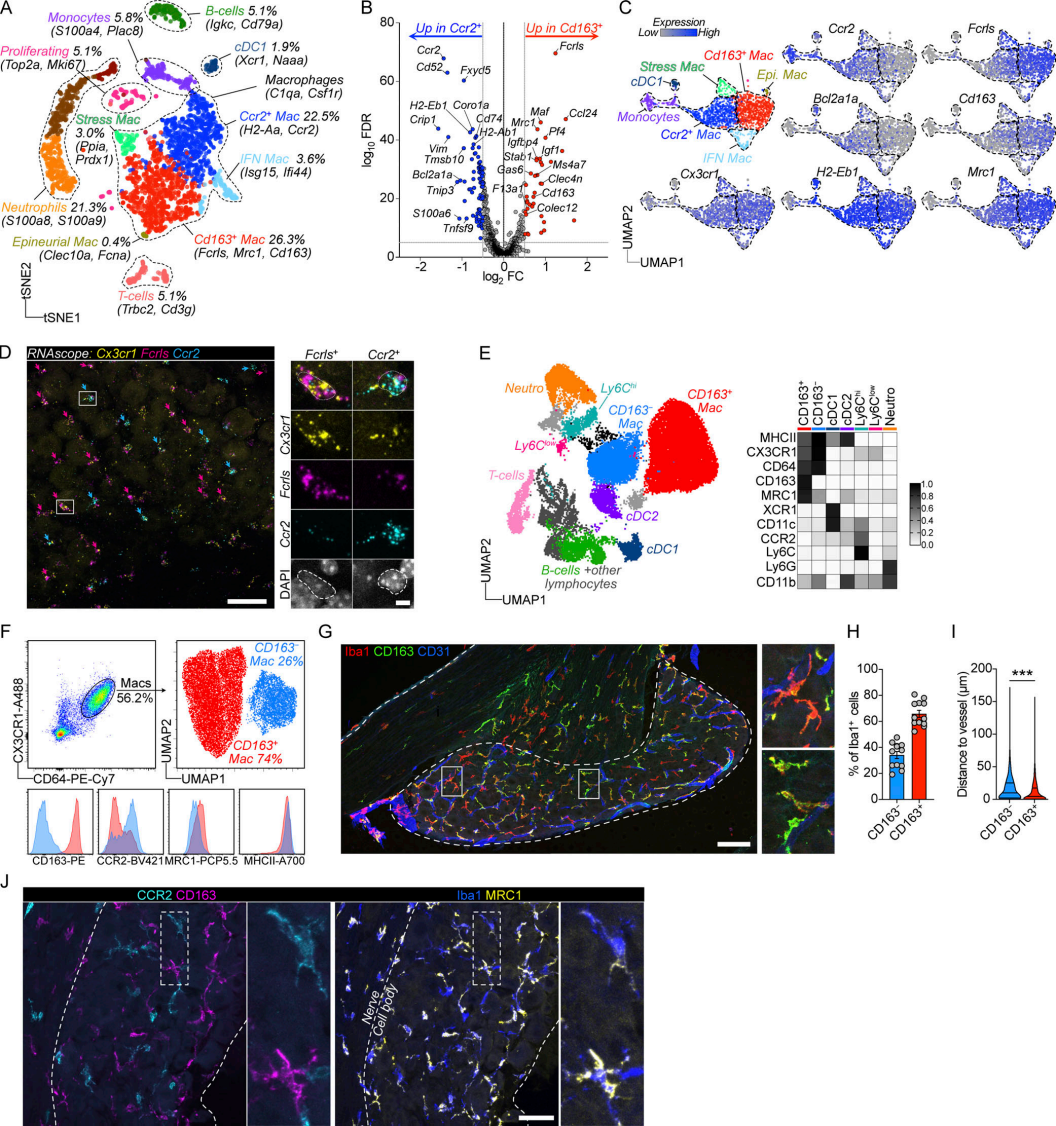
**DRG contains two molecularly distinct macrophage populations. (A)** scRNA-seq analysis of 2668 CD45^+^ DRG cells. *n* = 3 mice. **(B)** Differential gene expression between CD163^−^ and CD163^+^ macrophages using Venice algorithm. **(C)** UMAP of monocyte/macrophage/DC clusters and their expression of key transcripts. **(D)** Validation of *Fcrls* and *Ccr2* expression in separate subsets of *Cx3cr1*^*+*^ macrophages using RNAscope in DRG sections. Purple arrows indicate *Fcrls*^+^*Ccr2*^−^ cells and turquoise arrows indicate *Fcrls*^−^*Ccr2*^+^ cells. Images are representative of *n* = 3 mice. Scale bar, 50 and 5 μm (inset). **(E)** UMAP of live, CD45^+^ DRG cells analyzed by flow cytometry and expression heatmap of selected markers in all myeloid populations. *n* = 4 mice pooled. The experiment was performed three times. **(F)** Subclustering of CD64^+^CX3CR1^+^ macrophages from flow cytometry data. Histograms of key markers in resulting CD163^−^ and CD163^+^ macrophage clusters. **(G and H)** (G) Representative immunostaining and (H) quantification of Iba1^+^CD163^−^ and Iba1^+^CD163^+^ macrophages in DRG parenchyma. *n* = 12 mice. Scale bar, 100 μm. **(I)** Center-of-mass distance to nearest CD31^+^ blood vessel for CD64^+^CD163^−^ and CD64^+^CD163^+^ macrophages. Values are individual macrophages from *n* = 3 mice. Mann Whitney test. ***P < 0.001. **(J)** Immunostaining of CCR2 and MRC1 in DRG sections, displaying non-overlapping expression in CD163^−^ and CD163^+^ macrophages. Scale bar, 50 μm.

**Figure S3.**
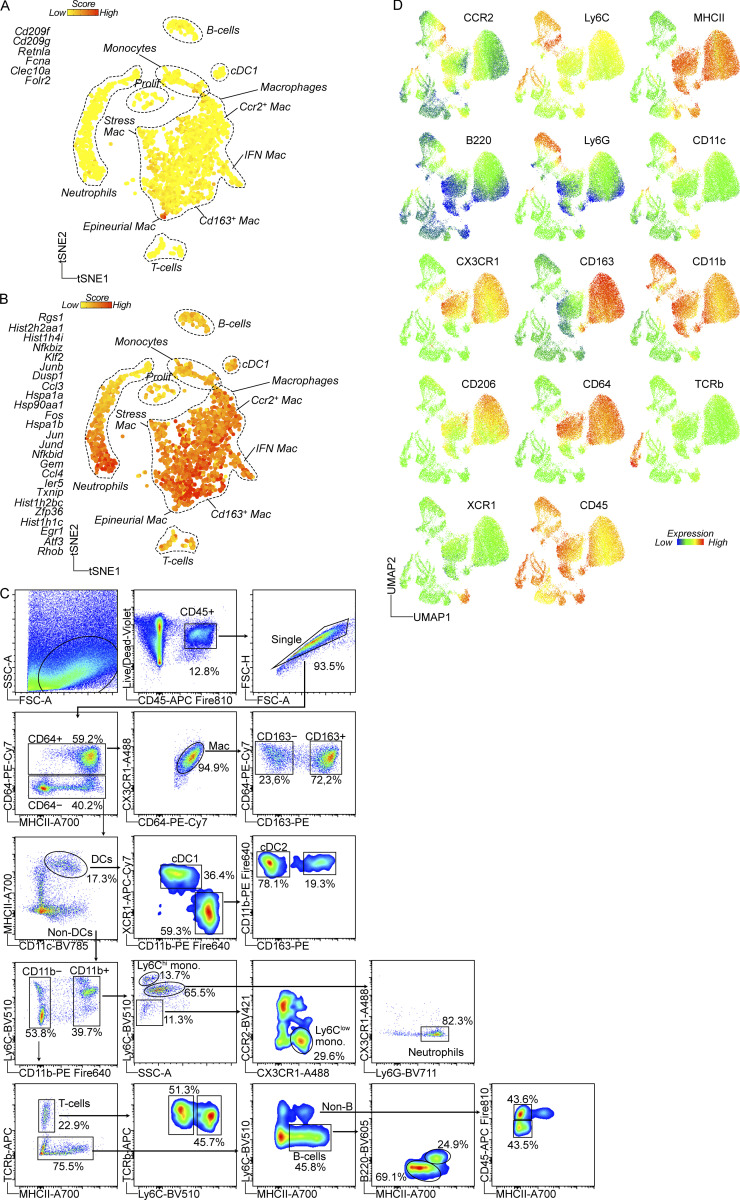
**Additional scRNA-seq and flow cytometry data of DRG immune cells. (A)** Epineurial macrophage signature (selected genes from Ydens et al. [2020]) across DRG immune cell types. **(B)** Ex vivo activation gene signature (25 genes identified in Marsh et al. [2022]) across DRG immune cell types. Induction of gene signature is most apparent in macrophages. **(C)** Conventional gating strategy to identify major immune cell populations in the UMAP. **(D)** Expression heatmap of all flow cytometry markers across UMAP clusters.

Our data thus far showed that macrophages in the DRG are predominantly perivascular and highly phagocytic, able to ingest a range of molecules crossing through ganglionic blood vessels. These results indicate specialization of the macrophage population at the blood–DRG interface, which prompted us to explore the DRG macrophage pool in greater detail. We used scRNA-seq using the 10× platform to profile the DRG immune landscape at steady state (*n* = 3 mice, 2,668 cells). Using unbiased clustering (Louvain) and dimensionality reduction (UMAP), we determined that the DRG was characterized by a heterogenous population of immune cells which included neutrophils (*S100a8*, *S100a9*), monocytes (*S100a4*, *Plac8*), B cells (*Igkc*, *Cd79a*), T cells (*Trbc2*, *Cd3g*), dendritic cells (DCs; *Xcr1*), but was numerically dominated by macrophages (*C1qa*, *Csf1r*, *Cx3cr1*; 59% of all cells; Fig. 4 A), which separated into two major clusters characterized by *Fcrls*, *Cd163*, and *Mrc1* (47.2% of macrophages) or *Ccr2* and *H2-Aa* expression (40.2% of macrophages), respectively. Three additional smaller macrophage clusters were present, one displaying interferon-regulated gene expression (*Isg15*, *Ifi44*, *Irf7*; 6.4% of macrophages), one expressing stress-induced genes (*Ppia*, *Prdx1*; 5.4% of macrophages), and one cluster expressing a signature of epineural macrophages (*Fcna*, *Cd209a*, *Clec10a*, *Folr2*; Ydens et al., 2020; Yim et al., 2022; 0.7% of macrophages; Fig. S3 A).

We next focused our analysis on the two largest macrophage clusters, as together they made up 87% of the total macrophage pool. Signs of ex vivo enzymatic digestion-induced gene expression (Marsh et al., 2022) were apparent, particularly in macrophages (Fig. S3 B), which included immediate early genes (*Fos*, *Jun*, *Atf3*, and *Rhob*). We thus removed these genes after differential gene expression (0.5 > log_2_FC; log_10_FDR > 5) and before gene ontology (GO) analysis. Differential gene expression demonstrated that the largest macrophage cluster (hereafter referred to as CD163^+^ macrophages) highly expressed *Fcrls*, encoding an Fc receptor-like glycoprotein with unknown function, as well as several phagocytic receptors including *Cd163*, *Mrc1*, and *Colec12* (Fig. 4, B and C). Consistently, GO analysis showed enrichment of receptor-mediated endocytosis (Table S2). *Maf* was also highly expressed in CD163^+^ macrophages, which is a transcription factor essential for perivascular macrophage survival and function (Moura Silva et al., 2021). *F13a1*, *Pf4*, and *Selenop*, which are all serum factors were also upregulated in CD163^+^ macrophages, similarly indicating an interaction with the blood. Consistently, platelet degranulation and regulated exocytosis were additional GO terms associated with CD163^+^ macrophages (Table S2). In addition, CD163^+^ macrophages expressed several chemokines (*Ccl7*, *Ccl8*, *Ccl12*, *Ccl24*, *Pf4*), resulting in enrichment of multiple terms related to chemotaxis (Table S2). The second cluster (hereafter referred to as CD163^−^ macrophages) specifically expressed *Ccr2* (Fig. 4, B and C), a chemokine receptor required for monocyte migration out of the bone marrow (Serbina and Pamer, 2006). Furthermore, *Ccr2* was recently identified as a marker of tissue macrophages that are constantly replaced by circulating monocytes (Dick et al., 2022). 29 ribosomal genes were expressed in this subset, resulting in enrichment of several GO terms related to protein translation (Table S3). After removal of ribosomal genes, GO terms related to neutrophil functions were enriched (*Lgals3*, *Adgre5*, *Anxa2*, and *Tlr2*), as well as mononuclear cell migration and type 2 immune responses (*Lgals3*, *Tnf*, *Tnfsf9*, and *Cd74*; Table S4). Furthermore, several MHCII genes (*H2-Aa*, *H2-Ab1*, *H2-Eb1*, and *H2-DMb1 Cd74*) were upregulated in CD163^−^ macrophages.

We next attempted to validate the in situ expression of the top two differentially expressed genes (*Fcrls* and *Ccr2*, Fig. 4, B and C). Using RNAscope, we confirmed expression of *Fcrls* and *Ccr2* in separate subsets of *Cx3cr1*^*+*^ macrophages, which were both located in the DRG parenchyma, interspersed between neuronal cell bodies (Fig. 4 D).

We next designed a flow cytometry panel to analyze the DRG myeloid landscape in greater detail. We used dimensionality reduction (UMAP) and unbiased clustering (Phenograph) based on 16 parameters (14 surface antigens, size, and granularity) combined with traditional gating (gating strategy in Fig. S3 C), and this revealed a similar distribution of macrophages, neutrophils, DCs, monocytes, and lymphocytes as in our scRNA-seq data (Fig. 4 E and Fig. S3 D). To investigate macrophage substructure, our panel included several pan-macrophage markers (CD11b, CX3CR1, and CD64), as well as potential subset-specific antibodies based on our scRNA-seq data (CD163, CCR2, MRC1, and MHCII). We visualized the DRG macrophage pool (CX3CR1^+^CD64^+^) separately, which assigned all macrophages into two major clusters in the resulting UMAP: CD163^+^CCR2^low^ and CD163^−^CCR2^hi^ (Fig. 4 F). MRC1 and MHCII expression provided additional separation between these subsets and were more highly expressed by CD163^+^ and CD163^−^ macrophages, respectively, which was consistent with our scRNA-seq data (Fig. 4 F). We next turned to immunohistochemistry (IHC) and confirmed the presence of CD163^−^ and CD163^+^ macrophages with similar frequencies as our flow cytometry data in the DRG tissue parenchyma (Fig. 4, G and H). While both macrophage subsets could be identified in both perivascular and non-perivascular locations (Fig. 4 G), CD163^+^ macrophages were on average found in closer contact with blood vessels than CD163^−^ macrophages (Fig. 4 I). Consistent with our flow cytometry data, we found that when CCR2 and MRC1 antibodies were applied to DRG sections, they preferentially labeled CD163^−^ and CD163^+^ macrophages, respectively (Fig. 4 J).

In summary, using scRNA-seq, multiparameter flow cytometry, and immunostaining, we identified two distinct macrophage subsets in the DRG, best defined by their differential expression of CD163.

### CD163^−^ and CD163^+^ macrophages have distinct life cycles

**Figure 5.**
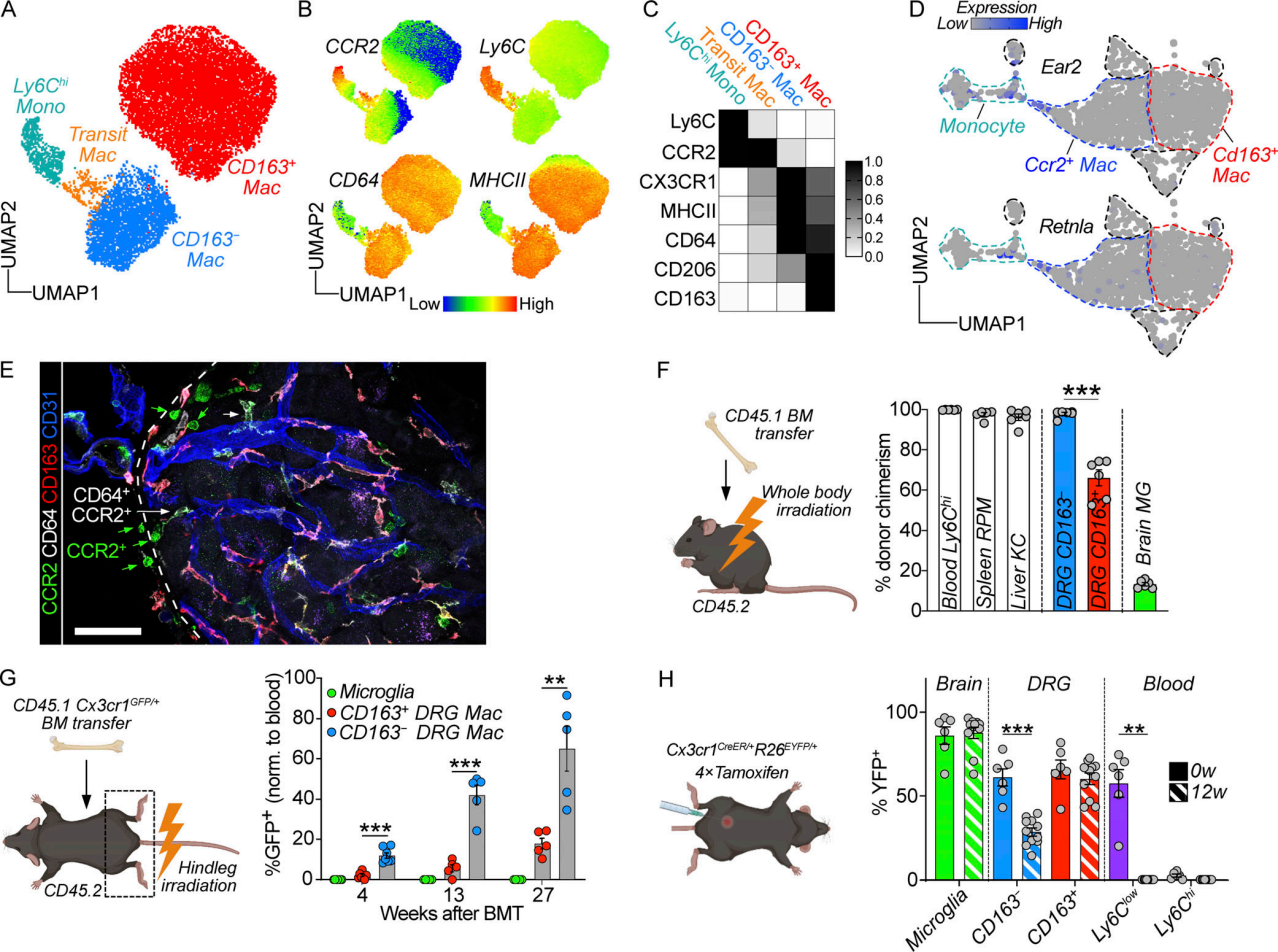
**The two DRG macrophage subsets display different turnover by monocytes. (A)** Subclustering of DRG monocyte/macrophage clusters from flow cytometry data presented in Fig. 4 E. *n* = 4 mice pooled. **(B)** Expression of indicated markers across clusters. **(C)** Expression heatmap of selected markers in indicated populations. **(D)** Expression of *Retnla* and *Ear2* (genes expressed in recently infiltrated monocytes, Sanin et al., 2022) within monocyte/macrophage clusters. **(E)** Immunolocalization of CCR2^+^ monocytes and CD64^+^CCR2^+^ macrophages adjacent to the DRG capsule. Scale bar, 50 μm. **(F)** Analysis of chimerism in CD45.2 mice 12 wk after whole body irradiation and i.v injection of 5 × 10^6^ CD45.1 bone marrow cells. Frequency of indicated cell populations that are of donor origin (CD45.1^+^), analyzed by flow cytometry. *n* = 6 mice, one experiment. Student’s unpaired *t* test. ***P < 0.001. RPM, red pulp macrophage. KC, Kupffer cell. MG, microglia. **(G)** CD45.2 mice received irradiation of only the hindlegs followed by i.v injection of 5 × 10^6^ bone marrow cells from CD45.1:*Cx3cr1*^GFP/+^ mice. Donor chimerism was assessed by immunostaining in tissue sections, analyzing GFP frequency in Iba1^+^ parenchymal microglia, Iba1^+^CD163^−^ or Iba1^+^CD163^+^ DRG macrophages at the indicated time points after irradiation. *n* = 7, 5, 5 mice. One experiment/time point. Multiple unpaired *t* tests with Holm-Sidak correction. **P < 0.01, ***P < 0.001. **(H)** *Cx3cr1*^CreER/+^*R26*^EYFP/+^ mice were given 4 × 2 mg tamoxifen injections i.p and YFP^+^ frequencies analyzed in indicated cell populations using flow cytometry after 72 h (0 w) or 12 wk (*n* = 6, 11 mice). Two experiments pooled. Sidak’s multiple comparisons test. **P < 0.01, ***P < 0.001. Mouse illustrations in F–H were created with https://BioRender.com.

Replenishment of tissue-resident macrophages by circulating monocytes is known to vary across and within tissues (Ginhoux and Guilliams, 2016), shaping macrophage phenotype and function (Blériot et al., 2020). High expression of *Ccr2* was recently identified in a subpopulation of tissue-resident macrophages across several organs that have a high turnover rate from circulating monocytes (Dick et al., 2022). We thus hypothesized that monocytes differentially contribute to the two identified DRG macrophage subsets during steady state, which could have implications on macrophage function. To gain further insight into the relationship between monocytes and CD163^−^ and CD163^+^ macrophages, we removed all lymphocyte, neutrophil, and DC clusters from our flow cytometry data and only reclustered cells expressing monocyte or macrophage markers. In the resulting UMAP, Ly6C^hi^ monocytes and CD163^−^ macrophages clustered closely and a new cluster of cells expressing intermediate levels of both monocyte and macrophage markers occupied the space in between (Fig. 5 A, referred to as “transitioning macrophages”). Ly6C and CCR2 downregulation as well as MHCII, CX3CR1, and CD64 upregulation characterized this transition (Fig. 5, B and C), which is consistent with the surface expression changes occurring in “monocyte-to-macrophage” conversion in the intestine (Bain et al., 2014; Tamoutounour et al., 2012). *Ear2* and *Retnla* were recently identified as early genes upregulated in monocytes that have recently infiltrated tissues and are committed to a macrophage fate (Sanin et al., 2022). Consistent with our flow cytometry data, we recorded expression of *Ear2* and *Retnla* in cells situated at the border of the CD163^−^ macrophage and monocyte clusters in the scRNA-seq UMAP (Fig. 5 D). In DRG tissue sections, CCR2^+^ cells with monocyte morphology were predominantly situated around the capsule and large veins/venules, suggesting monocyte infiltration and differentiation into CD64^+^CD163^−^CCR2^+^ macrophages occurring at this location (Fig. 5 E). Taken together, our flow cytometry and scRNA-seq data indicated that Ly6C^hi^ monocytes replenish CD163^−^ macrophages, while their contribution to CD163^+^ macrophages appeared to be limited.

To address experimentally if circulating monocytes differentially contributed to CD163^−^ and CD163^+^ macrophage populations, we turned to bone marrow chimeras. We first lethally irradiated CD45.2 mice and reconstituted them with CD45.1 bone marrow. Analysis of chimerism 12 wk later revealed complete replacement of circulating Ly6C^hi^ monocytes as well as splenic and liver macrophages (Fig. 5 F). Conversely, microglia only displayed 13% replacement by monocyte-derived macrophages, consistent with the well-described radio resistance of microglia and our own previous data (Lund et al., 2018). In the DRG, CD163^−^ macrophages were completely replaced by monocyte-derived cells, and while a majority of CD163^+^ were also donor-derived, 33% remained of host origin (Fig. 5 F). This experiment demonstrates that while Ly6C^hi^ monocytes are able to generate both CD163^−^ and CD163^+^ DRG macrophages, CD163^+^ macrophages display partial radio resistance.

**Figure S4.**
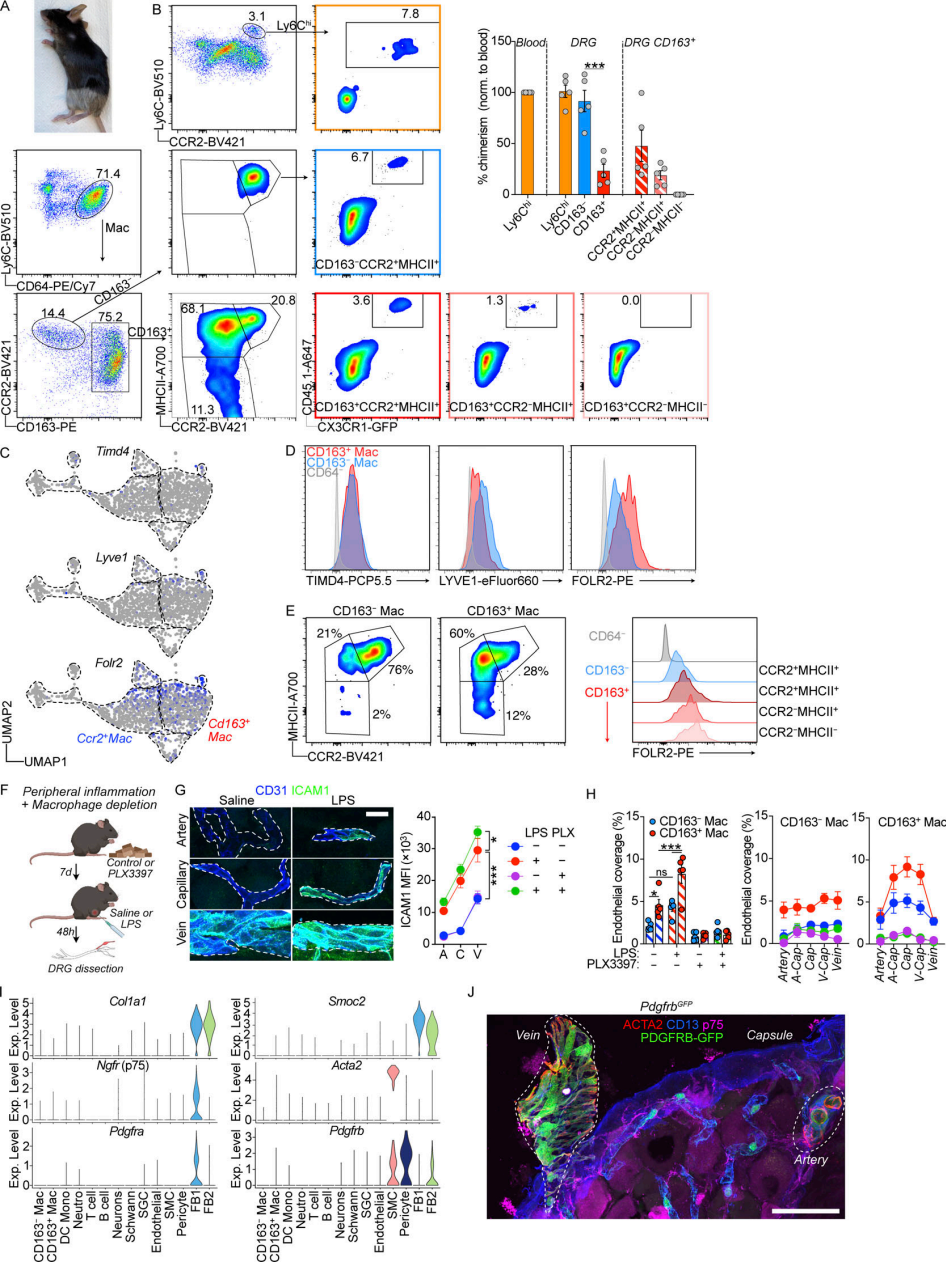
**Additional ontogeny data and response to peripheral inflammation by the two macrophage subsets. Additional analysis of DRG mural cells. (A)** Mouse fur at 27 wk after hindleg irradiation. **(B)** Flow cytometry–based analysis of hindleg-irradiated CD45.1:*Cx3cr1*^GFP/+^→ CD45.2 chimeric mice at 33 wk after irradiation. Chimerism was calculated as frequency of GFP^+^ cells within indicated cell subsets, normalized to blood GFP^+^ frequency. *n* = 5 mice. The experiment was performed once. Student’s unpaired *t* test. ***P < 0.001. **(C)** Expression of TLF genes (Dick et al., 2022) across monocyte/macrophage/DC clusters in scRNA-seq data from Fig. 4 A. **(D)** Expression of TLF markers by flow cytometry in indicated cell types using flow cytometry. *n* = 4 mice pooled. The experiment was performed twice. **(E)** FOLR2 expression in indicated macrophage subsets. **(F)** Experiment illustration for macrophage depletion and peripheral LPS challenge. Mice were fed control or PLX3397 chow (290 ppm) and injected i.p with 1 mg/kg LPS or saline. Created with https://BioRender.com. **(G)** Quantification of ICAM1 expression in CD31^+^ vessel segments. Two-way ANOVA with Tukey’s multiple comparisons test. *n* = 5 mice/group. The experiment was performed once. *P < 0.05, ***P < 0.001. Scale bar, 20 μm. A, artery; C, capillary; V, vein. **(H)** Quantification of endothelial coverage by Iba1^+^ perivascular macrophages, split into CD163^+^ and CD163^−^ subsets. Endothelial coverage was additionally analyzed based on which vessel segment macrophages contacted. *n* = 5 mice/group. The experiment was performed once. Sidak’s multiple comparisons tests. *P < 0.05, ***P < 0.001; ns, not significant. **(I)** mRNA expression of indicated fibroblast, SMC, and pericyte marker genes in the dataset used for CellChat (Avraham et al., 2020), presented in Fig. 7, A and B. **(J)** *Pdgfrb*^GFP/+^ mice label GFP^+^ACTA2^+^ SMCs covering arteries and veins. Scale bar, 50 μm.

To avoid the macrophage death and tissue inflammation that accompanies whole-body irradiation, we next set up tissue-protected chimeras. We irradiated only the hindlegs of CD45.2 mice and reconstituted them with CD45.1:*Cx3cr1*^GFP/+^ bone marrow (Fig. 5 G and Fig. S4 A), which after 4 wk resulted in ∼30% donor chimerism in the blood. We subsequently analyzed macrophage chimerism in the brain and DRGs over several time points up to 27 wk after irradiation. In contrast to our whole-body chimeras, microglia remained completely host-derived throughout the study period (Fig. 5 G). We next turned to analyzing DRGs and observed that over time Ly6C^hi^ monocytes differentially contributed to both CD163^−^ and CD163^+^ macrophages: at 4 wk, CD163^−^ macrophages were 12.1% donor derived, a number that rose to 42.0% after 13 wk and 65.1% after 27 wk (Fig. 5 G). For CD163^+^ macrophages, these numbers were 2.2% at 4 wk, 5.7% at 13 wk, and 18.0% at 27 wk (Fig. 5 G). In a parallel set of animals, we used flow cytometry on pooled DRGs to confirm the findings at the last time point (Fig. S4 B). When we separated CD163^+^ macrophages into three subpopulations based on CCR2 and MHCII expression, we noted a difference in the contribution of monocytes to these subclusters. Within the CD163^+^ gate, CCR2^+^MHCII^+^ and CCR2^−^MHCII^+^ macrophages were 47.8% and 19.0% donor derived, respectively, whereas CCR2^−^MHCII^−^ macrophages displayed 0.0% chimerism (Fig. S4 B). This indicated additional heterogeneity within the CD163^+^ population and suggested CD163^+^CCR2^−^MHCII^−^ macrophages could correspond to the recently described TLF macrophages (expressing *Timd4*, *Lyve1*, and/or *Folr2*), which are yolk-sac-derived self-sustaining macrophages populating many tissues (Dick et al., 2022). We thus explored the TLF markers and found only FOLR2 to be expressed at mRNA (Fig. S4 C) and protein levels (Fig. S4 D). Furthermore, FOLR2 expression negatively correlated with the bone marrow dependency found in our bone marrow chimeras (Fig. S4 E). This indicated that a subset of CD163^+^ could correspond to TLF macrophages.

To validate our results in a setting without irradiation, we made use of *Cx3cr1*^CreER/+^*R26*^EYFP/+^ mice, in which *Cx3cr1* expressing macrophages can be labeled by the administration of tamoxifen. 72 h after our tamoxifen regimen, on average, 85.9% of microglia were YFP^+^, a number that had not changed after 12 wk (87.6%; Fig. 5 H), which supports the well-described self-sustainability of microglia (Schulz et al., 2012; Hashimoto et al., 2013; Ajami et al., 2007; Ginhoux et al., 2010). Circulating Ly6C^hi^ monocytes displayed negligible labeling at 72 h and 12 wk. In the DRG, CD163^−^ and CD163^+^ macrophages displayed equal labeling 72 h after tamoxifen (61.1% versus 65.7% YFP^+^, respectively). 12 wk later, while CD163^+^ macrophages retained a similar level of YFP expression (60.2%), CD163^−^ macrophages had dropped to 28.5% YFP^+^ (Fig. 5 H).

Taken together, our data demonstrate that while CD163^−^ macrophages are constantly replenished from circulating monocytes, CD163^+^ macrophages are mostly self-sustained.

### Only CD163^+^ macrophages monitor the vasculature

**Figure 6.**
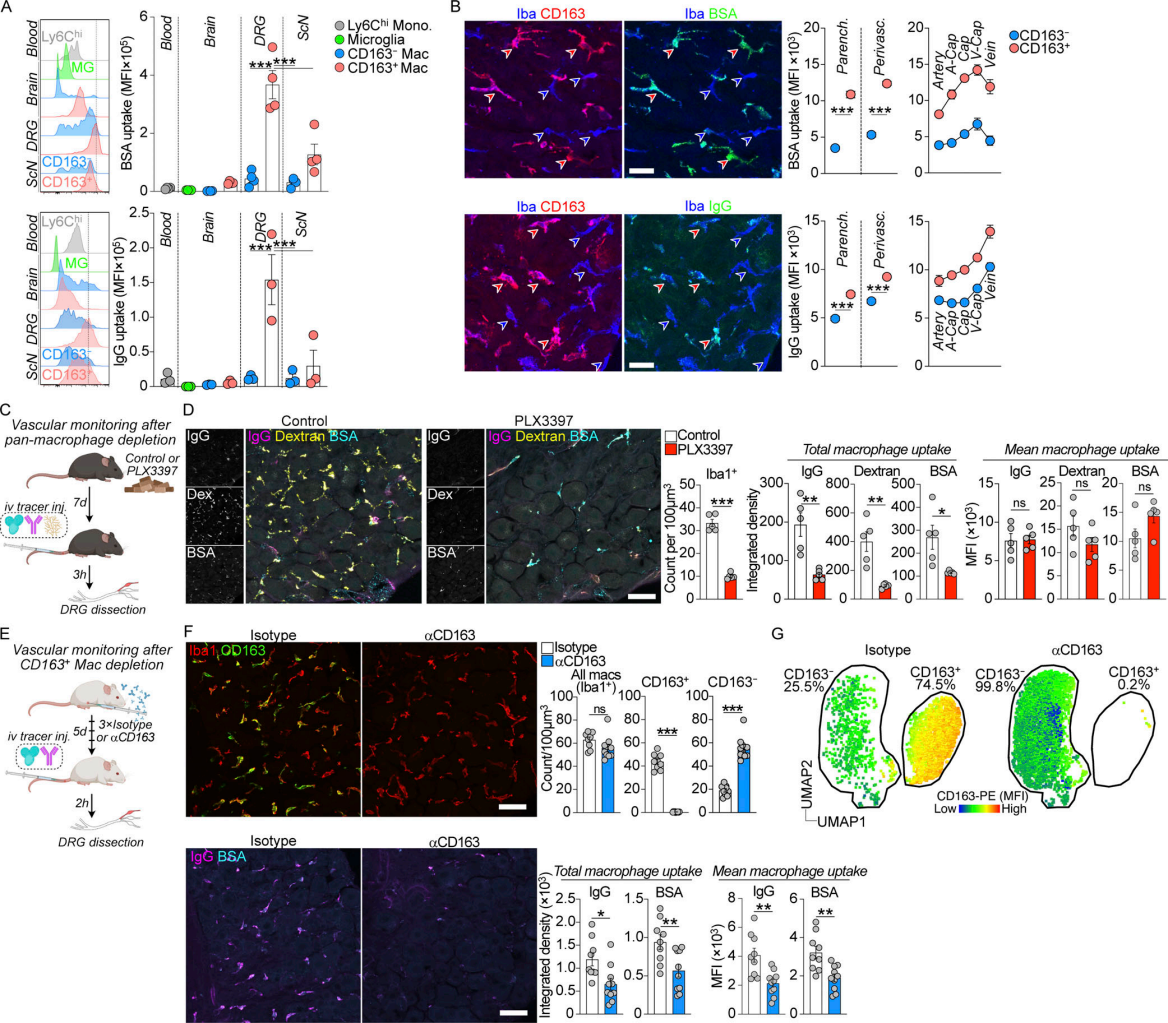
**Only CD163**^**+**^ **macrophages monitor the vasculature. (A)** Flow cytometry quantification of BSA-A647 (4 mg/kg, 1.5 h) and IgG-A647 (6 mg/kg, 2 h) uptake in indicated organs/cell subsets. Gated on CD11b^+^Ly6C^hi^ (monocytes), CX3CR1^hi^CD64^low^ (microglia), and CX3CR1^+^CD64^hi^ (macrophages). The experiment was performed two (IgG, *n* = 5 total) or three times (BSA, *n* = 8 total). Tukey’s multiple comparisons test. ***P < 0.001. **(B)** Machine-learning (DRGQuant)-based analysis of uptake of BSA-A647 (20 mg/kg, 1 h), goat anti rabbit IgG-A488 (4 mg/kg, 4 h) in CD163^−^Iba1^+^ and CD163^+^Iba1^+^ macrophages without (parenchymal) or with (perivascular) contact with vasculature. Perivascular macrophages were additionally analyzed based on which vessel segment they contacted. Student’s unpaired *t* test. ***P < 0.001. Scale bar, 25 μm. **(C)** Experiment schematic of tracer uptake in macrophage depleted mice using the CSF1R antagonist PLX3397 (290 ppm in chow). Created with https://BioRender.com. **(D)** Uptake of coinjected BSA-A647 (4 mg/kg), 70 kD dextran-TMR (10 mg/kg), and goat anti rabbit IgG-A488 (3 mg/kg) in Iba1^+^ macrophages 2 h 45 min after i.v injection. Depletion experiment was performed three times, uptake once. Student’s unpaired *t* test. *P < 0.05, **P < 0.01, ***P < 0.001. ns, not significant. Scale bar, 50 μm. **(E)** Experiment schematic of tracer uptake in CD163^+^ macrophage depleted mice using αCD163 or isotype control antibody (2.5 mg/kg, three injections separated by 48 h). Created with https://BioRender.com. **(F)** Quantification of macrophage subsets and uptake of i.v injected BSA-A488 (4 mg/kg) and goat anti human IgG-A647 (3 mg/kg) 2 h after i.v injection. *n* = 9, 10 mice. Two experiments pooled. Depletion experiment was performed three times, uptake twice. Student’s unpaired *t* test. *P < 0.05, **P < 0.01, ***P < 0.001. ns, not significant. Scale bar, 50 μm. **(G)** Flow cytometry UMAP of CD45^+^CD11b^+^Ly6C^−^CD64^+^CX3CR1^+^ macrophages after αCD163 mediated depletion (2.5 mg/kg, three injections separated by 48 h). *n* = 4 (isotype), 3 (αCD163).

We next assessed functional differences between the two DRG macrophage subsets. Given that CD163^+^ macrophages expressed several scavenger receptors and displayed a transcriptional signature associated with endocytosis, we hypothesized that CD163^+^ preferentially monitored DRG blood vessels. Consistent with this idea, using flow cytometry, we found that CD163^+^ macrophages phagocytosed more i.v injected BSA and IgG than CD163^−^ macrophages. This was particularly evident in the DRG, where we found the highest uptake of i.v injected tracers overall, but was also observed in ScN and brain macrophages (Fig. 6 A). We further confirmed higher tracer phagocytosis in CD163^+^ macrophages using DRG tissue sections, which additionally allowed us to distinguish macrophages based on their contact with the vasculature. Whereas CD163^+^ macrophages phagocytosed more tracer in every vessel segment, both CD163^−^ and CD163^+^ displayed similar uptake profiles along the arteriovenous axis, peaking in either v-caps or veins (Fig. 6 B). To assess vascular monitoring in the absence of CD163^+^ macrophages, we took two complementary approaches. First, we administered the CSF1R antagonist PLX3397 (Fig. 6 C), which resulted in 70–90% depletion of DRG macrophages (Fig. 6 D) and was accompanied by a reduction in the total uptake of i.v injected IgG, BSA, and dextran in DRG macrophages (Fig. 6 D). The mean uptake in macrophages was unchanged between groups. We next developed a depleting antibody to CD163 (Fig. 6 E). After three injections, we quantified DRG macrophages and found that this treatment resulted in complete removal of CD163^+^ macrophages from DRGs without affecting total macrophage numbers (Fig. 6 F). Analyzing depleted DRGs by flow cytometry and plotting the data in UMAP space revealed that all remaining DRG macrophages displayed an expression profile consistent with CD163^−^ identity (Fig. 6 G), indicating compensatory proliferation of surviving CD163^−^ macrophages as a result of CD163^+^ macrophage depletion. Consistent with a critical role of CD163^+^ macrophages for vascular monitoring in the DRG, we found that depleted mice displayed reduced total uptake and mean uptake of IgG and BSA in macrophages (Fig. 6 F). These results demonstrate that vascular monitoring is a function restricted to CD163^+^ macrophages.

### Peripheral inflammation drives arteriovenously zonated activation of CD163^+^ macrophages

We next investigated the effect of peripheral inflammation on macrophage-vasculature contact using LPS injection (Fig. S4 F). LPS led to robust activation of the endothelium in all vessel segments, as measured by upregulated ICAM1 expression (Fig. S4 G), which was accompanied by increased coverage of the vasculature by macrophages (Fig. S4 H). Mapping of macrophages along vessel segments demonstrated that endothelial coverage was also arteriovenously zonated, peaking in capillaries, and this was largely driven by increased contact with CD163^+^ macrophages (Fig. S4 H). These results demonstrate that peripheral inflammation drives zonated activation of CD163^+^ macrophages.

### IL34-producing pericytes interact with CD163^+^ macrophages

It is established that macrophages adopt tissue-specialized densities, identities, and functions by responding to signals in their local environment (Guilliams et al., 2020). To identify receptor–ligand interactions of biological importance for the two DRG macrophage subsets, we made use of recently published scRNA-seq profiles of all DRG cell types (Avraham et al., 2020) and performed CellChat analysis (Jin et al., 2021). We identified established cell–cell circuits such as neuronal to endothelial *Vegfb*-*Vegfr1* signaling (Kutcher et al., 2004) and endothelial to pericyte *Pdgfb*-*Pdgfrb* signaling (Armulik et al., 2010) as well as proposed interactions such as fibroblast to SGC *Col1a1/Col1a2-Sdc4* signaling (Vroman et al., 2023; Table S5), validating the ability of this approach to identify biologically relevant pathways.

**Figure 7.**
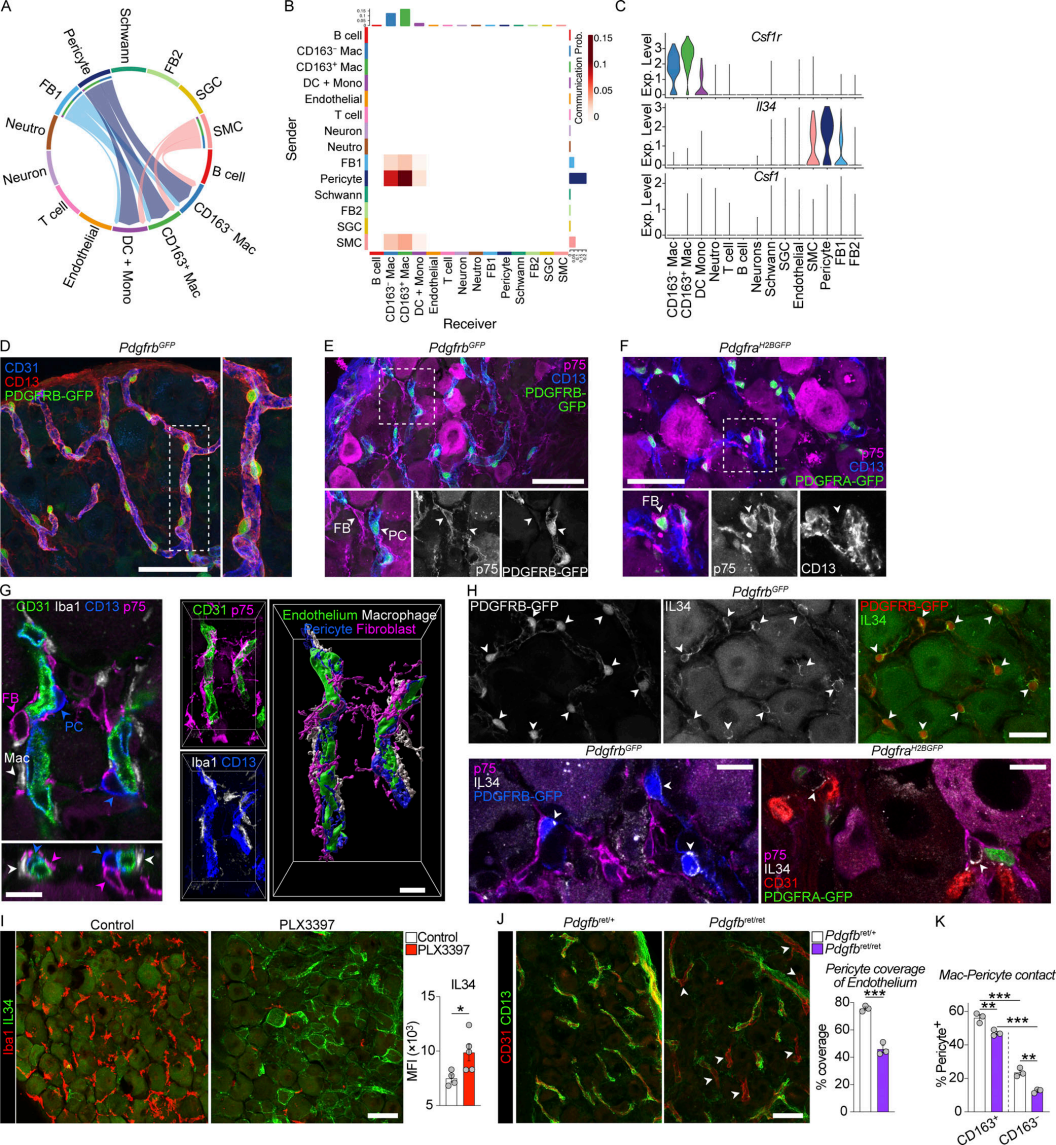
**Pericyte-macrophage interactions via the IL34-CSF1R axis. (A)** CellChat analysis of 1,592 naive DRG cells from a publicly available dataset (Avraham et al., 2020). Predicted cell–cell interactions via the IL34-CSF1R axis. **(B)** Heatmap visualizing communication probability between sender (y-axis) and receiver (x-axis) cells for the IL34-CSF1R axis using CellChat. **(C)** mRNA expression of indicated genes in the dataset used for CellChat. **(D and E)** Identification of capillary-wrapping CD13^+^GFP^+^p75^−^ pericytes (PC) in *Pdgfrb*^GFP^ mice. Scale bar, 50 μm. **(F)** Identification of p75^+^GFP^+^CD13^−^ fibroblasts (FB) in *Pdgfra*^H2GFP^ mice. Scale bar, 50 μm. **(G)** Z-stack and 3D rendering of Iba1^+^ macrophage, CD13^+^ pericyte, and p75^+^ fibroblast contacts on CD31^+^ DRG capillaries. Scale bars, 10 μm. **(H)** Localization of IL34 staining in GFP^+^ pericytes in *Pdgfrb*^GFP^ mice but absence from GFP^+^ fibroblasts in *Pdgfra*^H2GFP^ mice. Scale bars, 25 μm (top) and 10 μm (bottom). **(I)** IL34 staining in mice fed control or PLX3397 (290 ppm) chow for 7 d *n* = 5 mice/group. The experiment was performed twice. Student’s unpaired *t* test. *P < 0.05. Scale bar, 50 μm. **(J)** Analysis of coverage of CD31^+^ capillaries by CD13^+^ pericyte staining in *Pdgfb*^ret/ret^ and littermate *Pdgfb*^ret/+^ mice. Arrowheads indicate capillaries without pericyte coverage. Student’s unpaired *t* test. ***P < 0.001. Scale bar, 50 μm. **(K)** Analysis of contact between CD13^+^ pericytes and Iba1^+^CD163^+^ or Iba1^+^CD163^−^ macrophage subsets in *Pdgfb*^ret/ret^ and littermate *Pdgfb*^ret/+^ mice. *n* = 3 mice/group. Sidak’s multiple comparisons test. **P < 0.01, ***P < 0.001.

Next, we screened for receptor–ligand interactions predicted to be of high importance for CD163^+^ macrophages. Of note, we found *Il34-Csf1r* signaling from pericytes, smooth muscle cells (SMCs), and fibroblasts to macrophages (Fig. 7 A), with the strongest link predicted between pericytes and CD163^+^ macrophages (Fig. 7 B). Given the perivascular location of CD163^+^ macrophages and their capacity to self-maintain locally, we decided to explore this pathway further. Consistent with our results from CellChat, pericytes expressed the highest level of *Il34* during steady state, with lower levels detectable in SMCs and a subset of fibroblasts (expressing *Col1a1*, *Pdgfra*, and *Ngfr*, Fig. S4 I). *Csf1*, the alternative ligand for *Csf1r*, was not detectable in any cell type (Fig. 7 C). We next localized these cell types in the DRG using a combination of reporter mice and immunostaining. GFP^+^CD13^+^ pericytes were identified in *Pdgfrb*^GFP/+^ mice and were found to efficiently wrap around DRG capillaries (Fig. 7, D and E). SMCs (PDGFRB-GFP^+^ACTA2^+^) were identified around veins and arteries, as already described (Fig. S4 J). Fibroblasts were localized using *Pdgfra*^H2BGFP^ reporter mice and p75 (encoded by *Ngfr*) immunostaining (Fig. 7 F). Based on their mRNA profile (*Col1a1*, *Pdgfra*, *Ngfr*, *Smoc2*, Fig. S4 I) and immunoreactivity to p75 (Fig. 7, E and F), these cells likely correspond to endoneurial fibroblasts (Maniglier et al., 2022; Zhang et al., 2022), also referred to as tactocytes in the ScN (Malong et al., 2023). Both pericytes and fibroblasts were found to interact with macrophages on capillaries (Fig. 7 G). By immunostaining, IL34 protein was localized to the cell membrane of pericytes but was absent in fibroblasts (Fig. 7 H). Moreover, IL34 protein expression was increased in the DRG after pan-macrophage depletion using PLX3397, suggesting an IL34-driven feedback loop to sustain macrophage numbers (Fig. 7 I). These data indicated that pericyte-derived IL34 was important for maintaining DRG macrophages. In WT mice, we found a closer interaction between pericytes and CD163^+^ macrophages than their CD163^−^ counterparts (Fig. 7 K), which is consistent with a more important role of the IL34–CSF1R axis for CD163^+^ macrophages, as predicted by CellChat. Finally, we investigated *Pdgfb*^ret/ret^ mice, which have a pericyte deficiency in the CNS (Armulik et al., 2010). In the DRG, *Pdgfb*^ret/ret^ mice also presented with reduced endothelial coverage by pericytes (Fig. 7 J), which resulted in decreased pericyte-CD163^+^ macrophage contact in *Pdgfb*^ret/ret^ mice (Fig. 7 K). Our data thus identifies pericyte-derived IL34 as a potential source of survival factors sustaining the CD163^+^ macrophage network.

### Perivascular CD163^+^ macrophages are conserved in human DRGs

**Figure 8.**
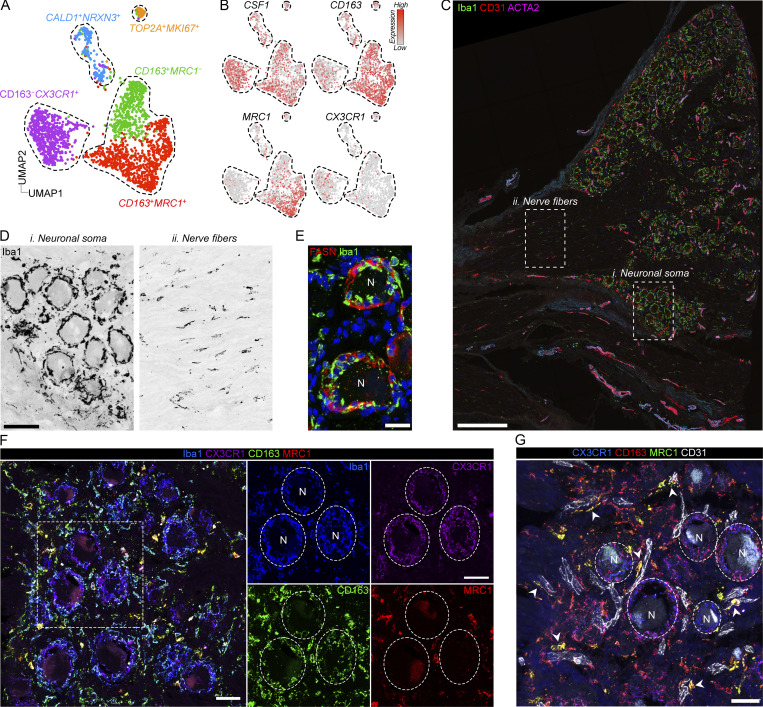
**Perivascular CD163**^**+**^ **macrophages are conserved in human DRGs. (A)** UMAP of 2,098 subclustered macrophage nuclei from five human DRG donors (Avraham et al., 2022). **(B)** Expression of key indicated genes across clusters. **(C)** Iba1 immunostaining of a large human DRG section visualizing regional differences in macrophage density. Scale bar, 500 μm. **(D)** Boxed areas in C visualizing the difference in macrophage distribution between neuronal soma-rich and nerve fiber-rich areas. Scale bar, 100 μm. **(E)** Immunostaining of Iba1^+^ perineuronal macrophages and FASN^+^ SGCs. While distinct, both cell types cluster around neuron cell bodies. Scale bar, 25 μm. **(F)** Immunostaining showing the perineuronal localization of CX3CR1^+^Iba1^+^ macrophages and the interstitial localization of Iba1^+^CD163^+^MRC1^+^ macrophages. Scale bars, 50 μm. **(G)** Immunostaining showing perivascular location of CD163^+^MRC1^+^ macrophages. Scale bar, 50 μm. N, neuron.

We next addressed whether CD163^−^ and CD163^+^ macrophages were also present in human DRGs. We again made use of a published snRNA-seq dataset of human DRGs (Avraham et al., 2022) that contained 2,098 macrophages (clusters expressing *CSF1R*). To investigate macrophage substructure, we clustered only macrophages, resulting in five distinct clusters (Fig. 8 A). The largest cluster comprised 38.0% of all macrophages and was characterized by expression of *CD163*, *MRC1*, *F13A1*, *STAB1*, and *COLEC12* (Fig. 8 B), thus displaying considerable overlap in gene expression with the CD163^+^ macrophage population identified in mouse DRGs. GO analysis revealed enrichment of biological processes such as receptor-mediated endocytosis (*CD163*, *COLEC12*, *TFRC*, *MRC1*, *STAB1*), endothelial tube morphogenesis (*STARD13*, *RBPJ*), and iron/heme metabolism (*HMOX1*, *FXIIIA*, *FTL*, *BLVRB*; Table S6), indicating that this subset (*CD163*^+^*MRC1*^+^) had a function related to the vasculature, just as we had observed in the mouse. An additional cluster (*CD163*^+^*MRC1*^−^) accounting for 21.2% of macrophages also expressed *CD163* but lacked most of the defining markers of the *CD163*^+^*MRC1*^+^ macrophages, including *F13A1*, *COLEC12*, and *MRC1*. No genes were specifically upregulated in this cluster. A third cluster accounted for 29.6% of macrophages and expressed *C3*, *OXR1*, and *KCNIP1* highly, but lacked *CD163*. Differential gene expression also identified *CX3CR1* as a defining marker for this subset (*CD163*^−^*CX3CR1*^+^). While this subset displayed similar frequency as CD163^−^ macrophages in the mouse, it did not overlap in the gene expression profile. In addition, two smaller clusters expressing markers related to regulation of neuronal function (CALD1^+^NRXN3^+^, 8.7%), as well as proliferation (TOP2A^+^MKI67^+^, 2.5%) were also evident (Fig. 8 A). CD163^+^ macrophages are thus conserved in human DRGs and display similar gene expression profiles and putative function.

To validate the presence of the major identified macrophage subsets in human tissues, we again turned to DRG sections from human organ donors (Table S1). We first used the pan-macrophage marker Iba1, which as in the mouse revealed a much higher accumulation of macrophages in the neuronal soma-rich region of DRGs compared with the nerve-rich region (Fig. 8, C and D). In the neuronal soma-rich region, macrophages formed dense aggregates around the neuronal cell bodies but were distinct from FASN^+^ SGCs (Fig. 8 E), which also inhabit this space. In addition, macrophages populated the interstitial space between the neuronal cell bodies. We next applied subset-specific antibodies to DRG sections and observed that CX3CR1 expression was virtually restricted to macrophages surrounding the neuronal soma (Fig. 8 F). Conversely, we found that CD163^+^MRC1^+^ and CD163^+^MRC1^−^ macrophages were preferentially located in the interstitium rather than perineuronally (Fig. 8 F). Costaining of CD31^+^ endothelial cells further revealed that CD163^+^MRC1^+^ were localized in close contact with endothelial cells (Fig. 8 G).

**Figure S5.**
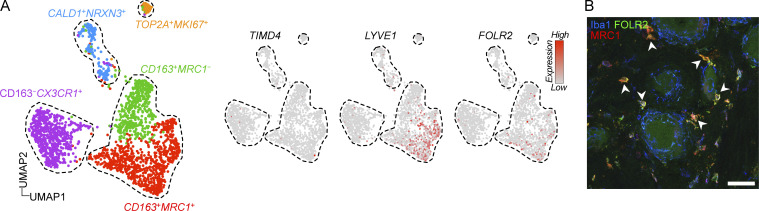
**Expression of TLF genes in human DRG macrophages. (A)** UMAP of 2,098 subclustered macrophage nuclei from five human DRG donors (Avraham et al., 2022) and expression of TLF markers across clusters. **(B)** FOLR2 expression by immunostaining localized to Iba1^+^MRC1^+^ interstitial macrophages (arrowheads) and absence from Iba1^+^MRC1^−^ perineuronal macrophages. Scale bar, 50 μm.

To investigate the ontogeny of CD163^+^MRC1^+^ macrophages, we further explored their transcriptional profile and found that *LYVE1* and *FOLR2* were specifically expressed in this subset (Fig. S5 A). CD163^+^MRC1^+^ macrophages thus expressed the core signature (*CD163*, *MRC1*, *F13A1*, *FOLR2*, and *LYVE1*) of macrophages isolated from the embryonic yolk sac as well as yolk-sac-derived macrophages in adult human tissues (Dick et al., 2022). We also confirmed specific expression of FOLR2 on the protein level in CD163^+^MRC1^+^ using immunostaining (Fig. S5 B). Although conclusive evidence is lacking, our data suggest that CD163^+^MRC1^+^ macrophages are of yolk sac origin in human DRGs.

In summary, we identify CD163^−^CX3CR1^+^ neuron-associated macrophages which display limited transcriptional and spatial overlap with mouse DRG macrophage subsets. By contrast, CD163^+^MRC1^+^ macrophages are transcriptionally, anatomically, and ontogenically conserved in human DRGs.

## Discussion

The vasculature is organized into networks of arteries, veins, and interconnected capillaries. While this general structure is shared between all organs, transcriptional and anatomical heterogeneity in vascular identity is well characterized (Potente and Mäkinen, 2017; Trimm and Red-Horse, 2022), giving rise to organotypic vascular beds with distinct functional properties. The vasculature of the CNS is characterized by several features to maintain high barrier integrity: low level of transcytosis, constitutive expression of tight and adherens junctions, absence of fenestrae, and almost complete coverage of endothelial cells by astrocytes and pericytes (Zhao et al., 2015). The BNB shares several but not all of these features (Malong et al., 2023) and is recognized as the second most restrictive vascular system in the body (Ubogu, 2020). In contrast to the BBB and BNB, it is well documented that the blood vessels supplying the sensory ganglia are more permeable to circulating molecules than those supplying the axons (Jacobs et al., 1976; Kobayashi and Yoshizawa, 2002; Olsson, 1968; Arvidson, 1979). However, a cohesive mechanism for this phenomenon has been lacking. We here combined scRNA-seq data with tracer injections to anatomically and functionally map the DRG arteriovenous tree, identifying impermeable CLDN5^+^PLVAP^−^CAV1^−^ arteries/a-caps situated close to the nerve fibers and CLDN5^−^PLVAP^+^CAV1^+^ veins/v-caps underlying the capsule.

Regional differences in tight junction expression, including CLDN5, between neuronal soma-rich regions and fiber-rich regions in the DRG have been reported (Lux et al., 2019; Hirakawa et al., 2004). However, experimental evidence suggests CLDN5 only regulates permeability to small molecules as CLDN5 mutant mice display increased permeability only to molecules <0.8 kD at the BBB (Nitta et al., 2003) and does not affect basal permeability to 10–70 kD dextrans in skin, trachea, skeletal muscle, or heart (Richards et al., 2022). We instead focused our attention on PLVAP-expressing cells, a protein that is restricted to the diaphragm of endothelial fenestrae, transendothelial channels, and caveolar vesicles (Stan et al., 1999, 2004). Despite several descriptions of DRG endothelial fenestrae in the literature (Jacobs et al., 1976; Anzil et al., 1976; Arvidson, 1979; Kobayashi and Yoshizawa, 2002), we observed that <0.1% of the endothelial lumen was covered with fenestral openings. By comparison, 12.8% of the luminal surface of the glomerular endothelium of the kidney is covered by fenestrae (Bulger et al., 1983). Based on this finding, we find it unlikely that endothelial fenestrae contribute significantly to basal permeability of the DRG endothelium. However, we do not exclude that fenestrae serve other critical purposes important for DRG physiology, such as chemosensation of the body’s internal milieu, similar to sensory circumventricular organs (Miyata, 2015), as previously proposed (Devor, 1999). While fenestrae were limited, as much as 10% of the endothelial cytoplasm in v-caps contained caveolar vesicles. It has been proposed that caveolae and fenestrae are interchangeable structures (Satchell and Braet, 2009). However, we excluded this possibility in the DRG endothelium based on the presence of fenestrae in *Cav1* mutant mice. Furthermore, using these mice, we functionally validated the importance of caveolar vesicles for basal permeability of the DRG endothelium. In addition, we identify the lipid transporter MFSD2A (Nguyen et al., 2014; Ben-Zvi et al., 2014), an inhibitor of caveolar assembly (Andreone et al., 2017; Wang et al., 2020b), as a potential regulator of caveolar transcytosis also in the DRG.

After peripheral nerve damage, macrophages accumulate not only at the site of injury but also around axotomized neuronal cell bodies in the DRG (Kalinski et al., 2020; Peng et al., 2016; Niemi et al., 2013), a process that promotes axon regeneration (Kwon et al., 2013; Niemi et al., 2013; Feng et al., 2023) as well as the development of neuropathic pain (Yu et al., 2020). Most studies of DRG macrophages have been guided by this and analogous findings, which have placed a heavy focus on macrophage-sensory neuron crosstalk in the understanding of DRG macrophage biology (Gheorghe et al., 2022). We here propose that an additional critical function of DRG macrophages is to interact with the vasculature. Several of our experiments support this claim: CD163^+^ macrophages displayed a vasculature-associated transcriptional profile, made close contact with endothelial cells, received survival signals from endothelial-associated pericytes, rapidly phagocytosed circulating macromolecules, and increased vessel coverage in response to circulating endotoxin. A primary function of CD163^+^ macrophages could thus be to limit the enhanced permeability of the blood–DRG barrier, a function that is described for perivascular macrophages in the cochlea (Zhang et al., 2012), skin (He et al., 2016), and more recently in the ScN (Malong et al., 2023). Depletion of macrophages in all these organs results in vessel hyperpermeability. Similarly, CD163^+^ macrophages located in close proximity to fenestrated blood vessels in the area postrema of the brain sequester blood proteins (Willis et al., 2007). However, we did not observe increased leakage of injected tracers into the DRG parenchyma following macrophage depletion, instead arguing for an active role of DRG macrophages in sampling of the circulation.

Lyve1^lo^MHCII^hi^ and Lyve1^hi^MHCII^lo^ interstitial macrophages were recently described in lung, fat, heart, and dermis that preferentially associated to nerve fibers or blood vessels, respectively (Chakarov et al., 2019). Lyve1^hi^MHCII^lo^ expressed MRC1 and CD163 and were previously identified in the aortic wall and found to regulate arterial tone by degrading collagen (Lim et al., 2018). Perivascular macrophages with similar gene expression (including *Lyve1*, *Mrc1*, *Cd163*, and *Maf*) and high phagocytic capacity have also been described in white adipose tissue, intestines (Moura Silva et al., 2019, 2021), and brain (Drieu et al., 2022). Thus, the CD163^+^ vascular-monitoring subset that we have identified in our study is likely shared across multiple tissues. In the DRG, MRC1 expression has been used to identify “M2-macrophages,” which resolve osteoarthritis- (Raoof et al., 2021) or chemotherapy-induced pain by producing IL-10 (Singh et al., 2022) or inflammatory pain by transferring mitochondria to sensory neurons (van der Vlist et al., 2022). Whether the MRC1^+^ macrophages identified in these reports correspond to the CD163^+^ vasculature-monitoring subset identified in our study remains to be explored.

In an effort to link the ontogeny of the DRG macrophage subsets to their phenotype and function (Blériot et al., 2020), we also addressed their turnover from circulating monocytes. This revealed that CD163^+^ macrophages operated without substantial input from monocytes and thus may at least partly correspond to TLF macrophages, which are embryonically derived self-maintaining macrophages that express *Cd163* (Dick et al., 2022). By contrast, CD163^−^ macrophages (expressing CCR2) were almost completely replaced by monocytes over 27 wk. This finding is supported by parabiosis experiments, which found the presence of parabiont-derived macrophages in the DRG parenchyma in naive mice (Guimarães et al., 2023). Furthermore, our findings also agree with the study by Dick et al, which described tissue-resident CCR2^+^ macrophages that were almost completely replaced by circulating monocytes (Dick et al., 2022). It should be noted, however, that the study by Chakarov et al. found that both Lyve1^lo^MHCII^hi^ and Lyve1^hi^MHCII^lo^ interstitial macrophages were replenished by monocytes during steady state (Chakarov et al., 2019).

Finally, when investigating human DRGs, we observed a similar arteriovenous zonation as in mouse as well as the presence of CD163^+^ perivascular macrophages. Our study thus identifies two mechanisms that are likely conserved in humans that regulate blood–DRG barrier permeability: caveolar transcytosis and phagocytosis by CD163^+^ macrophages. Both mechanisms could be pharmacologically targeted to reduce extravasation of circulating molecules, or alternatively co-opted to deliver drugs into the DRG parenchyma (Marchetti et al., 2019; Kiseleva et al., 2018; Skytthe et al., 2020). This could be desirable in sensory ganglionopathies (Amato and Ropper, 2020), which include paraneoplastic and autoimmune conditions, infections, platinum-based chemotherapy (Gupta and Bhaskar, 2016; Dzagnidze et al., 2007), and likely also fibromyalgia (Goebel et al., 2021; Martínez-Lavín, 2021; Krock et al., 2023).

## Materials and methods

### Mice

All mice were either purchased from approved vendors or bred and maintained under specific pathogen–free conditions at Karolinska Institutet or Uppsala University in accordance with national animal care guidelines. All animal experiments were approved by the appropriate ethical review board (Stockholms djurförsöksetiska nämnd). C57Bl/6 mice were purchased from Charles River (C57BL/6J) or bred locally (C57BL/6NTac). The following strains were originally purchased from the Jackson Laboratory or acquired through collaboration and bred at Karolinska Institutet: CD45.1 (Jax 002014), *Cav1*^−/−^ mice (Jax 007083; Razani et al., 2001), *Cx3cr1*^gfp^ (Jax 005582, gift from C. Gerlach, Karolinska Institutet; Jung et al., 2000), *Cx3cr1*^CreER^ (Jax 020940; Yona et al., 2013) and R26R-EYFP (Jax 006148; Srinivas et al., 2001), *Pdgfrb*^GFP/+^ (Gong et al., 2003) and *Pdgfra*^H2BGFP^ (JAX 007669, gift from M. Gennander, Karolinska Institutet; Hamilton et al., 2003). *Cldn5*(BAC)-eGFP mice (Laviña et al., 2018) and *Pdgfb*^ret/ret^ (Lindblom et al., 2003) were bred at Uppsala University. All bred strains were on a C57Bl/6 background, and where applicable, littermate control animals were used as controls. In experiments with *Cav1*^−/−^ mice, age- and sex-matched C57BL/6NTac mice (bred in the same room) were used as controls. BALB/cAnNRj mice were purchased from Janvier and used for the scRNA-seq experiment and CD163 depletion experiments. Both male and female mice were used, and experiments were started when mice were 7 wk or older.

### Generation of chimeric anti-mouse CD163 depleting antibody

Anti-mouse CD163 depleting antibody or isotype control was generated by recombinant expression of a rat anti-mouse CD163 Fab region (clone E10B10; Etzerodt et al., 2012, 2019) or a rat anti-diphtheria toxoid Fab region fused with murine IgG2a Fc region (Vazquez-Lombardi et al., 2018) in ExpiCHO expression system (Thermo Fisher Scientific) according to the manufacturer’s instructions. Expressed chimeric IgG was purified using protein A purification columns and quantified using absorbance on a nanodrop2000 system. Lastly, chimeric IgG was assessed essentially as endotoxin-free (<0.1 EU/mg protein) using the HEK-Blue LPS bioassay (Invivogen)

### In vivo studies

#### Macrophage depletion using PLX3397

PLX3397 was formulated into A04 standard diet (Safe Nutrition Service) at 75 ppm or 290 ppm and was administered ad libitum for 7 or 21 consecutive days. An identical diet without PLX3397 was used as control.

#### CD163^+^ macrophage depletion

CD163 depleting antibody or isotype control was diluted in PBS and administered i.p at 2.5 mg/kg three times, each injection separated by 48 h. Experiments were performed 24 h after the last injection.

#### Tamoxifen administration

Tamoxifen (T5648; Sigma-Aldrich) was resuspended in corn oil (C8267; Sigma-Aldrich) and administered i.p to *Cx3cr1*^CreER^*R26*^EYFP^ mice at 1 mg/10 g body weight, four times over a 5-day period.

#### Whole-body irradiation chimera

CD45.2 mice were irradiated with 9.5 Gray using an X-RAD 320 irradiation source (0.95 Gray/min) with a 20 × 20 cm irradiation field and reconstituted the same day with 5 × 10^6^ CD45.1 bone marrow cells by tail vein injection. Tissue chimerism was analyzed 12 wk later.

#### Hindleg bone marrow chimera

Irradiation of only hindlegs was accomplished by maintaining CD45.2 mice under isofluorane anesthesia and placing the body outside the field of irradiation (the irradiation source is equipped with a lamp to visualize the 20 × 20 cm irradiation field). Mice were reconstituted the same day with 5 × 10^6^ bone marrow cells from CD45.1/CD45.2:*Cx3cr1*^gfp/+^ or CD45.1/CD45.1:*Cx3cr1*^gfp/+^ mice by tail vein injection and analyzed at 4, 13, 26, or 33 wk.

#### Tracer injections

Anesthetic cream was applied to the tail 20 min prior to i.v injections. Tracers were injected via the tail vein at 4 μl/g body weight. Doses and circulation times are summarized in figure legends for each experiment. The following tracers were used: Goat anti Rabbit IgG-A488 (A11008; Thermo Fisher Scientific), Goat anti Human IgG-A647 (109-605-003; Jackson Immuno), BSA-A488 (A13100; Thermo Fisher Scientific), BSA-A647 (A34785; Thermo Fisher Scientific), 3 kD dextran-TMR (D3308; Thermo Fisher Scientific), 70 kD dextran-TMR (D1818; Thermo Fisher Scientific), 500 kD dextran-FITC (D7136; Thermo Fisher Scientific), and 2,000 kD dextran-FITC (D7137; Thermo Fisher Scientific). Mice were subsequently sacrificed and transcardially perfused with PBS only and immediately dissected (flow cytometry analysis) or PBS followed by 4% formaldehyde (IHC analysis) followed by 24 h after fixation in 4% formaldehyde.

#### LPS injections

Mice were injected i.p with LPS (1 mg/kg; 0111:B4; Condrex, Serotype) or saline vehicle and sacrificed 48 h later.

### Whole mount and optical clearing

DRG staining and optical clearing were performed according to Hunt et al. (2022) using a modified version of the iDISCO protocol (Renier et al., 2014). Briefly, DRGs dissected with the dorsal/ventral roots and peripheral nerve attached were washed and permeabilized in PBS and 0.2% Triton X-100 for 3 h, rinsed in PBS and 0.2% Tween-20 (PTw). DRGs were then incubated 72 h with primary antibodies diluted in PTw. After thorough washing in PTw, DRGs were incubated 48 h with secondary antibodies diluted in PTw protected from light. Finally, DRGs were washed in PTw before tissue clearing. Stainings were performed in 0.5 ml Eppendorf tubes on a tube rotator. DRGs were either imaged directly (CLDN5-GFP) or optically cleared to improve signal depth. Optical clearing was performed in 50-ml glass flasks at room temperature (RT) by first dehydrating DRGs in increasing concentrations of tetrahydrofuran in distilled water: 50%, 70%; 80%, 2 × 100% (10 min/solution) followed by refractive index matching in dibenzyl ether (DBE; 2 × 10 min). Cleared tissues were mounted in DBE in custom-made 3D-printed image chambers according to Hunt et al. (2022) and imaged using a confocal microscope.

### Immunostaining

Standard methods for immunostaining were applied. Tissues were collected from animals perfused with 4% formaldehyde followed by direct dissection and no post-fixation or post-fixation for 24 h followed by dissection. The different fixation protocols were chosen based on downstream staining protocols. Tissues were embedded in OCT and sectioned using a cryostat at 25–40 μm onto SuperFrost Plus glass slides. Sections were stored at −20°C until staining. For staining, sections were allowed to thaw at RT for 1 h, washed in PBS for 20 min, and blocked with 3% donkey serum in PBS + 0.2% Triton X-100 for 30–60 min. Tissues were then incubated with primary antibodies (Table S7), diluted in 0.2% Triton X-100, overnight at 4°C. After 3 × 10 min washes in PBS, secondary antibodies (Thermo Fisher Scientific or Jackson ImmunoResearch), diluted in PBS, were applied and sections incubated for 2 h at RT. After another round of washing, sections were mounted using ProLong gold (Thermo Fisher Scientific). If not otherwise stated, 3D images shown in figures were made using Imaris software, and 2D images presented in figures are maximum intensity projections of acquired z-stacks.

### RNAscope

RNAscope was performed according to the manufacturer’s protocol for RNAscope Multiplex Fluorescent Detection Reagents v2 (323110; ACD). Briefly, slides were washed in 1× PBS for 5 min and baked in the HybEZ Oven for 30 min at 60°C. A 15-min fixation in cold 4% PFA was done prior to dehydration with 50%, 70%, and twice with 100% ethanol for 5 min at RT. Slides were then dried at RT and H_2_O_2_ (322335; ACD) was applied so that the tissue was covered. This incubation was done for 10 min at RT and the slides were washed in MilliQ water. The slides were transferred into a 1× target retrieval solution (322000; ACD) at 100°C and kept in this solution for 5 min, after which they were washed in MilliQ and 100% ethanol. A circle was drawn around the tissue using a ImmEdge Pen (H-4000; Vector laboratories). The slides were left to dry at RT overnight. Protease III (322337; ACD) was applied for 30 min at 40°C in the HybEZ Oven. The slides were washed in MilliQ water and a solution of 1× probes was applied for 2 h at 40°C in the HybEZ Oven. The 1× probe solution was prepared by diluting F*crls*-C2 (441231-C2; ACD) and *Cx3cr1*-C4 (314221-C4; ACD) 50 times in 1× *Ccr2*-O1 (501681; ACD). The slides were washed using 1× wash buffer (310091; ACD) and the amplification reagents AMP1, AMP2, and AMP3 were applied for 30, 30, and 15 min, respectively, at 40°C with washes in wash buffer after each incubation. HRP-C1 was applied for 15 min at 40°C after which the slides were washed in wash buffer and incubated with Opal dye 570 (OP-001003; Akoya) diluted 1:1,500 in TSA buffer (322809; ACD) for 30 min at 40°C. Upon another wash in wash buffer, the HRP blocker solution was applied for 15 min at 40°C. The same steps were repeated for HRP-C2 with Opal 690 (OP-001006; Akoya) and HRP-C4 with Opal 520 (OP-001001; Akoya) diluted 1:3,000. After the final wash, spectral DAPI was applied to the slides for 30 s. The slides were mounted with ProlongGold mounting media.

### Confocal imaging

Z-stack images were acquired using a Zeiss LSM800 laser-scanning confocal microscope equipped with four lasers (405, 488, 561, and 640 nm).

### STED

Super-resolution STED imaging was performed using a STEDYCON (Abberior Instruments) equipped with excitation lasers at 488, 561, and 640 nm and a STED laser at 775 nm. Deconvolution was performed on all STED images using Huygens software.

### TEM

Mice were perfused with 2.5% glutaraldehyde and 1% formaldehyde in 0.1 M phosphate buffer, pH 7.4, followed by post-fixation in the same solution (>24 h). Following fixation, the DRGs were rinsed in 0.1 M phosphate buffer prior to post-fixation in 2% osmium tetroxide in 0.1 M phosphate buffer, pH 7.4, at 4°C for 2 h. DRGs were then stepwise dehydrated in ethanol followed by acetone and resin embedded in LX-112 (Ladd). Ultrathin sections (∼80–100 nm) were prepared using an EM UC 7 (Leica) and contrasted with uranyl acetate followed by lead citrate. The sections were examined using a Hitachi HT7700 transmission electron microscope (Hitachi High-Technologies) at 80 kV and digital images were acquired using a 2kx2k Veleta CCD camera (Olympus Soft Imaging Solutions).

### SEM

Following rinsing in 0.1 M phosphate buffer and MilliQ, DRGs were subjected to stepwise dehydration using ethanol and prior to critical point drying in an EM CPD 030 (Leica). The DRGs were finally mounted on aluminum pins using double-sided carbon adhesive tabs and platinum coated using a Q150T ES (Quorum). The DRGs were analyzed using an Ultra 55 field emission scanning electron microscope (Zeiss) at 3 kV using the SE2 detector.

### Image analysis (Imaris)

Imaris software was used to quantify marker expression across DRG vessel segments. PLVAP and CLDN5-GFP were quantified in sections stained with CD31 and ACTA2 and vessels assigned as arteries (ACTA2^+^, diameter >10 μm), capillaries (ACTA2^−^, diameter <10 μm), or veins (ACTA2^+^ on DRG surface, diameter >10 μm). The zonated expression of PLVAP and CLDN5 in the DRG vasculature was subsequently utilized to quantify MFSD2A and CAV1 expression by mean fluorescence intensity (MFI) across vessel segments using the following criteria: arteries (same as above), a-caps (ACTA2^−^, PLVAP^−^, or CLDN5^+^ diameter <10 μm), and v-caps (ACTA2^−^, PLVAP^+^, or CLDN5^−^, diameter <10 μm). MFI expression was calculated for each vessel segment and normalized to %max for each animal.

### Image analysis (DRGQuant)

Images were analyzed using the DRGQuant pipeline described previously (Hunt et al., 2022). In brief, UNET (Ronneberger et al., 2015, *Preprint*) models were trained to identify vasculature, macrophages, pericytes, satellite glial cells, neuronal-rich regions of the DRG, fiber-rich regions of the DRG, endothelial cells (TEM), and vascular lumen (TEM). Model outputs were then run through macros in FIJI (Schindelin et al., 2012) that segmented structures using connected component analysis in CLIJ (Haase et al., 2020). Macrophage identities were classified as follows: DRG macrophage (≥30% vol within the neuronal soma-rich region), fiber macrophage (non-DRG macrophage with ≥30% vol in the fiber-rich region), parenchymal macrophage (surface area of <10 μm^2^ in contact with blood vessels), and perivascular macrophage (surface area >10 μm^2^ in contact with blood vessels). A FIJI macro was written for semi-automated segmentation of the arteriovenous axis with the following workflow: 2D projections of vascular/endothelial cell markers were annotated by an expert as vein, v-cap, capillary, a-cap, or artery. Annotations were converted into 2D masks, which were then applied to the 3D vasculature map identified via UNET. Perivascular macrophages were then classified based on the vessel type they were most closely associated with. Macrophage morphology as well as fluorescent intensities were quantified using ImageJ’s 3D region of interest manager (Ollion et al., 2013). For quantifications, the following definitions were used: percent volume was calculated by total volume of objects/total volume of tissue. For morphological quantifications, elongation was defined as the ratio of the major radius of the ellipsoid to the second radius of the ellipsoid, and sparseness the ratio between the volume of the ellipsoid and the volume of the object. A script was written in Python that concatenated all data tables generated in FIJI. For identification of endothelial vesicles (caveolae) in TEM images, a stardist (Schmidt et al., 2018) model was trained. A FIJI macro was written that used the UNET output in combination with the stardist output to isolate only vesicles present in the endothelial cells. Single vesicles were defined by a diameter of <100 nm and fused vesicles were defined by a diameter >100 nm. For all datasets, a summary image highlighting all classified objects was generated and visually inspected to ensure the quality of the analysis.

### Generation of single-cell suspensions

Mice were euthanized with pentobarbital (338327; APL) overdose i.p. When applicable, blood was collected from the right ventricle prior to perfusion into an EDTA- or heparin-coated syringe. Mice were perfused with ice-cold PBS and neural tissues dissected into cold PBS. For analysis by flow cytometry, a 5-mm coronal slice of the right forebrain was used, both sciatic nerves and ∼40 pooled cervical, thoracic, and lumbar DRGs. Neural tissues were digested in 2 mg/ml Collagenase I (17100-017; Gibco), 5 mg/ml Dispase II (D4693; Sigma-Aldrich), and 0.5 mg/ml DNAse I (11284932001; Roche) at 37°C for 30 min (Brain) or 40 min (DRGs and ScN). Myelin was removed using 38% Percoll (GE17-0891-02; Sigma-Aldrich) and the cells were subsequently washed and resuspended in PBS. 200 μl blood was lysed in ACK buffer (A1049201; Gibco) and centrifuged. The pellet was resuspended in PBS and used for staining.

### Flow cytometry

Flow cytometry data were acquired using a Cytek Aurora equipped with violet (405 nm), blue (488 nm), and red (640 nm) lasers. The following antibodies and stains were used: CCR2-BV421 (clone 475301, 747963; BD), Ly6C-BV510 (clone HK1.4, 128033; Biolegend), B220-BV605 (clone RA3-6B2, 103243; Biolegend), Ly6G-BV711 (clone 1A8, 127643; Biolegend), CD11c-BV785 (clone N418, 117336; Biolegend), CX3CR1-A488 (clone SA011F11, 149021; Biolegend), CX3CR1-BV785 (clone SA011F11, 149029; Biolegend), CD163-PE (clone TNKUPJ, 12-1631-80; Thermo Fisher Scientific), CD64-PE/Cy7 (clone X54-5/7.1, 139313; Biolegend), MRC1-PCP5.5 (clone C068C2, 141716; Biolegend), CD11b-PEFire640 (clone M1/70, 101279; Biolegend) TCRb-APC (clone H57-597, 109212; Biolegend), MHCII-A700 (clone M5/114.15.2, 107622; Biolegend), XCR1-APC/Cy7 (clone ZET, 148223; Biolegend), CD45-APC-Fire810 (clone 30F11, 103173; Biolegend), CD45.1-A647 (clone A20, 110720; Biolegend), CD45.2- BV785 (clone 104, 109839; Biolegend), and LIVE/DEAD Violet dead cell stain kit (L34955; Thermo Fisher Scientific). Flow cytometry data was analyzed using Flowjo 10 software. Dimensionality reduction and cluster identification were performed using the UMAP and Phenograph packages, respectively.

### scRNA-seq

Single-cell suspensions of DRG cells from BALB/cAnNRj mice were blocked with FcR blocking solution (l No.130-092-575; Miltenyi Biotec) and stained with anti-CD45:PE (103105; Biolegend). Additionally, TotalSeq anti-mouse Hashtag antibodies were used to label individual samples (155803, 155805, 155809; Biolegend). The samples were strained through a 35-micron filter. DAPI was added to the cell solution to exclude dead cells. CD45^+^DAPI^−^ cells were sorted on a BD Influx sorter. The sorted cells were pooled into a single lane on the 10x Genomics Chromium Single Cell 3′ v3 system. Library preparation and sequencing were performed at the SciLifeLab sequencing facility, Solna, using an Illumina HiSeq 2500 at 61,006 reads/cell. The 10X CellRanger output files (barcodes, feature, and count matrix) were analyzed using Seurat in R studio. The filtering criteria were set to include cells with >200 and <5,000 genes as well as <5% mitochondrial reads. The data was normalized and scaled using the Seurat functions (NormalizeData and ScalaData). The three samples were demultiplexed using the HTODemux function. Variable features were found using the *vst* method in the FindVariableFeatures function with nfeatures set to 2,000 and were used to compute the principal components using RunPCA. K-nearest and shared nearest-neighbor analyses were computed using FindNeighbors with 15 dimensions. Graph-based clustering was conducted using the Louvain algorithm in the FindClusters function with the resolution set at 0.5 (informed by clustertree produced by Clustree). RunUMAP was used to visualize the clusters. Annotation was done using the SingleR package to compare transcriptomes in the ImmunoGen database and based on known cell type markers (Aran et al., 2019). For visualization and differential gene expression analysis, data were exported to Bioturing Browser 3.

### Endothelial cell enrichment and quantitative PCR (qPCR)

Single-cell suspensions were prepared from liver, lung, kidney, brain, and DRGs by enzymatic digestion at 37°C using the following enzymes and incubation times. Liver, small piece of one lobe (1 mg/ml Collagenase I 17100-017; Gibco, 1 mg/ml; Collagenase II LS004176; Worthington, 5 mg/ml Dispase II D4693; Sigma-Aldrich, and 7.5 μg/ml DNAse I 11284932001, 30 min; Roche), lung, one lobe (1 mg/ml; Collagenase II, 2.5 mg/ml Collagenase IV LS004188; Worthington, and 15 μg/ml DNAse I, 50 min), kidney, one (2 mg/ml; Collagenase I and 7.5 μg/ml DNAse I, 45 min), forebrain (2 mg/ml; Collagenase I, 0.5 mg/ml DNAse I, 30 min), and DRGs, ∼40 (2 mg/ml; Collagenase I, 5 mg/ml Dispase II, 0.5 mg/ml DNAse I, 45 min). Single-cell suspensions were resuspended in FACS buffer and labeled with CD31 microbeads (130-097-418; Miltenyi) and enriched on MS columns (130-042-201; Miltenyi) according to the manufacturer’s instructions. Cells were pelleted and lysed in RLT buffer. RNA was isolated using the RNeasy Micro Kit (74004; Qiagen) and reverse transcribed using iScript cDNA synthesis kit (1708890; Bio-Rad) according to manufacturer instructions. qPCR was performed on a C1000 Touch thermal cycler equipped with a CFX384 detection module (Bio-Rad) with SYBR Green Master Mix reaction (4367659; Bio-Rad) and the following PCR primer sequences (Sigma-Aldrich): *Gapdh* (F: 5′-TGT​AGA​CCA​TGT​AGT​TGA​GGT​CA-3′, R: 5′-AGG​TCG​GTG​TGA​ACG​GAT​TTG-3′), *Pecam1* (F: 5′-ACG​CTG​GTG​CTC​TAT​GCA​AG-3′, R: 5′-TCA​GTT​GCT​GCC​CAT​TCA​TCA-3′), *Slc2a1* (F: 5′-CAG​TTC​GGC​TAT​AAC​ACT​GGT​G-3′, R: 5′-GCC​CCC​GAC​AGA​GAA​GAT​G-3′), *Slc7a5* (F: 5′-CTT​CGG​CTC​TGT​CAA​TGG​GT-3′, R: 5′-TTC​ACC​TTG​ATG​GGA​CGC​TC-3′), *Mfsd2a* (F: 5′-AAA​GAC​ACG​CAA​AAT​GCT​TAC​CT-3′, R: 5′-AAT​GAA​GGC​ACA​GAG​GAC​GTA​GA-3′), *Gpihbp1* (F: 5′-AGG​GCT​GTC​CTC​CTG​ATC​TTG-3′, R: 5′-GGG​TCC​GCA​TCA​CCA​TCT​T-3′), *Slco2a1* (F: 5′-ATT​AAG​GTC​TTC​GTG​CTT​TGT​CA-3′, R: 5′-GTA​GGC​ACT​GTA​GAG​CAA​CTG-3′).

### Human tissue

Human lumbar DRG were obtained from organ donors through a collaboration with Transplant Quebec. All procedures were approved by and performed in accordance with the ethical review board at McGill University (McGill University Health Centre REB 2019-4896). Familial consent was obtained for each subject. Human DRGs were delivered frozen and prior to use and were fixed in 4% formaldehyde for 3–6 h followed by cryoprotection in 30% sucrose for 3–5 d at 4°C. DRGs were embedded in OCT and sectioned at 12–50 μm using a cryostat. Donor details are listed in Table S1.

### Analysis of publicly available data

#### Bioturing Browser 3

Bioturing Broswer 3 (Le et al., 2020, *Preprint*) was used to analyze the following deposited studies: E-MTAB-8077 (Kalucka et al., 2020) (mouse multiorgan endothelial cells, raw counts) and GSE139103 (Avraham et al., 2020) (mouse DRG cells, raw counts). Gene sets for ex vivo activation scores were retrieved from Marsh et al. (2022). For all studies, clustering was performed using the Louvain method. Differential gene expression was performed using the Venice method (Vuong et al., 2020, *Preprint*). Signature scores were calculated as the sum of expressions normalized by the total count (Pont et al., 2019). If applicable, batch correction was performed using canonical correlation analysis (Stuart et al., 2019).

Enrichment scores for BBB and peripheral endothelium-specific genes based on bulk RNA-seq of brain vs. heart, lung, liver, and kidney endothelial cells were retrieved from Munji et al. (2019).

#### R studio

snRNA-seq data of human DRG cells from five individuals (GSE169301) (Avraham et al., 2022) were downloaded and analyzed using Seurat (version 4.3.0.1) in R studio (Hao et al., 2021). The filtering criteria were set to include cells with >200. The data were normalized and scaled using the Seurat functions (NormalizeData and ScalaData). The samples were integrated using the functions SelectIntegrationFeatures and FindIntegrationAnchors and IntegrateData. Variable features were found using the vst method in the FindVariableFeatures function with nfeatures set to 2,000 and were used to compute the PCAs using RunPCA. K-nearest and shared nearest-neighbor analyses were computed using FindNeighbors with 16 dimensions. Graph-based clustering was conducted using the Louvain algorithm in the FindClusters function with the resolution set at 0.5 (informed by clustertree produced by Clustree). RunUMAP was used to visualize the clusters. The Seurat object was subsetted to macrophages by selecting clusters that expressed *CSF1R* (three clusters). The new object was reanalyzed with the functions ScaleData, RunPCA, FindNeighbors (10 dimensions), FindClusters (resolution 0.2), and RunUMAP. To analyze endothelial cells, the cluster expressing *PECAM1* was selected and reanalyzed with the functions ScaleData, RunPCA, FindNeighbors (17 dimensions), FindClusters (resolution 0.2), and RunUMAP. For visualization and differential gene expression analysis, data were exported to Bioturing Browser 3.

#### CellChat

Two control samples of mouse DRGs from GSE139103 were downloaded and analyzed with the Seurat package (version 4.3.0.1). Briefly, the filtering criteria were set to include cells with >200 and <5,000 genes as well as <5% mitochondrial reads. The functions ScaleData, FindVariableFeatures (method set to vst with 2,000 features), RunPCA, FindNeighbors (15 dimensions), FindClusters (resolution 0.5), and RunUMAP were run. Within the macrophage cluster, cells with *Fcrls* expression > 0 were annotated “CD163+” and otherwise “CD163−.” The R package CellChat (version 1.6.1) was utilized to computationally predict cell-to-cell communication (Jin et al., 2021). The Seurat object of the reanalyzed mouse DRG data was converted to a CellChat object. The analysis was done using the standard CellChat workflow (including the functions: indentifyOverExpressedGenes, identifyOverExpressedInteractions, computeCommunProb, filterCommunication, computeCommunProbPathway, and aggregateNet). Default options were used for all functions.

### Online supplemental material

Fig. S1 shows macrophage flow cytometry data related to Fig. 1 and endothelial zonation data related to Fig. 2. Fig. S2 shows tracer experiments, TEM analyses, and Cav1/Mfsd2a qPCR and IHC data related to Fig. 3. Fig. S3 shows macrophage scRNA-seq and flow cytometry data related to Fig. 4. Fig. S4 shows flow cytometry data from hindleg irradiated chimera and analysis of TLF genes/proteins related to Fig. 5, peripheral inflammation experiment related to Fig. 6, and mural cell mRNA and IHC data related to Fig. 7. Fig. S5 shows human snRNA-seq and IHC data related to Fig. 8. Table S1 lists information on human organ donors included in IHC analysis. Table S2 lists GO terms enriched in *Cd163*^+^ mouse macrophages. Table S3 lists GO terms enriched in *Ccr2*^+^ mouse macrophages. Table S4 lists GO terms enriched in *Ccr2*^+^ mouse macrophages after ribosomal genes are removed. Table S5 shows results from CellChat analysis. Table S6 lists GO terms enriched in human *CD163*^+^*MRC1*^+^ macrophages. Table S7 lists antibodies used for IHC. Video 1 shows perivascular macrophage identification in DRG. Video 2 shows identification of blood vessel segments in DRG.

## Supplementary Material

Table S1 lists information on human organ donors included in IHC analysis.

Table S2 lists GO terms enriched in *Cd163*^+^ mouse macrophages.

Table S3 lists GO terms enriched in *Ccr2*^+^ mouse macrophages.

Table S4 lists GO terms enriched in *Ccr2*^+^ mouse macrophages after ribosomal genes are removed.

Table S5 shows results from CellChat analysis.

Table S6 lists GO terms enriched in human *CD163*^+^*MRC1*^+^ macrophages.

Table S7 lists antibodies used for IHC.

## Data Availability

Data supporting the findings of this research article are available upon request to the corresponding author. The scRNA-seq data generated during this study are available through Gene Expression Omnibus accession number GSE246168.
